# Can maternal characteristics improve due date prediction without ultrasound? Development and internal validation of a machine-learning prediction model in 600 000 pregnancies in Sweden

**DOI:** 10.1136/bmjopen-2026-120970

**Published:** 2026-10-23

**Authors:** Gilson Dutra, Mikael Elinder, Oscar Erixson

**Affiliations:** 1 Department of Economics, Uppsala University, Uppsala, Sweden; 2 Institute for Housing and Urban Research, Uppsala University, Uppsala, Sweden

**Keywords:** Pregnancy, Maternal medicine, Machine Learning, ULTRASONOGRAPHY, Fetal medicine

## Abstract

**Abstract:**

**Objectives:**

To assess whether estimated due dates based on Naegele’s rule can be improved by incorporating routinely collected maternal characteristics into a non-parametric machine learning model, particularly in settings where ultrasound is unavailable.

**Design:**

Population-based retrospective cohort study; development and internal validation of a machine-learning prediction model.

**Setting:**

Nationwide register data from Sweden.

**Participants:**

603 861 singleton pregnancies recorded in the Swedish Medical Birth Register between 2008 and 2017. We restricted the sample to spontaneous singleton births with complete information on last menstrual period, ultrasound dating and maternal characteristics and further limited the primary analysis to pregnancies in which both Naegele’s rule and ultrasound-based gestational age at delivery fell between 37 and 42 completed weeks.

**Primary and secondary outcome measures:**

The primary outcome was prediction accuracy of the date of delivery. Performance was evaluated using exact-day accuracy, accuracy within ±1 and ±7 days, mean squared error and root mean squared error. Machine-learning predictions were compared with those obtained using Naegele’s rule and ultrasound-based dating under identical inclusion criteria.

**Results:**

The machine-learning model improved prediction accuracy over Naegele’s rule across all evaluated measures, while ultrasound-based dating remained more accurate overall. The model reduced between 25% and 45% of the accuracy gap between Naegele’s rule and ultrasound. Subgroup-specific models provided little additional improvement. Among second-time mothers, however, adding gestational duration from the previous pregnancy yielded markedly greater gains and produced prediction accuracy comparable to, and on some measures slightly better than, ultrasound.

**Conclusions:**

Incorporating maternal characteristics into a regression-forest model improved due date prediction compared with Naegele’s rule. These improvements may be relevant in settings where ultrasound is unavailable but the routinely collected information and basic digital infrastructure required to apply the model are available. The approach is not intended to replace ultrasound-based dating in routine clinical care. Further research is needed to assess performance in other populations and to evaluate potential implications for clinical decision-making. The model was developed and internally validated in Swedish register data and requires external validation before use as a decision support tool elsewhere.

**STRENGTHS AND LIMITATIONS OF THIS STUDY**

- This study uses a large, population-based dataset of more than 600 000 pregnancies from a nationwide medical birth register.
- This study applies a transparent machine learning approach with a predefined train–validation–test split to minimise overfitting.
- This study evaluates Naegele’s rule, ultrasound-based dating and machine learning predictions under identical inclusion criteria, ensuring comparability across methods.
- This study is restricted to spontaneous singleton term births (37–42 weeks), which improves internal validity but limits generalisability to preterm, post-term or medically managed pregnancies.
- This study relies on routinely collected maternal characteristics and complete-case analysis, which supports scalability but may limit applicability in settings with missing or lower-quality data.

## Introduction

 Obstetric care and management have improved dramatically over the last century, with substantial increases in the survival of both newborns and mothers. A key advancement has been the introduction of ultrasound examinations early in pregnancy, which provide accurate estimates of gestational age and the expected due date by relating fetal size to established reference charts.^1–4^ Accurate dating is important for reducing perinatal risks, including stillbirth and infant mortality,^5–8^ and it is crucial for planning obstetric care as well as for providing parents with reliable information about the expected timing of birth.^9^

Despite the benefits of ultrasound-based dating, access to ultrasound remains limited in many parts of the world. Although the WHO^10^ recommends that all pregnant women undergo an ultrasound examination, recent data suggest that roughly two-thirds of pregnant women globally do not have access to routine ultrasound screening.^11^ As a result, large parts of the world’s population do not benefit from the improvements in gestational age determination provided by ultrasound.

In settings without ultrasound, gestational age and expected due date are typically estimated using Naegele’s rule, a simple calendar-based method that adds 280 days to the reported first day of the last menstrual period. Naegele’s rule has been used for nearly two centuries^12^ and remains widely applied early in pregnancy, even in high-income countries. However, it performs less well than ultrasound-based dating when predicting the timing of delivery.^13 14^

Both Naegele’s rule and standard ultrasound-based dating approaches rely on a common reference gestational duration of 280 days. In practice, however, predictions based solely on the last menstrual period often contain systematic errors arising from recall error, variation in menstrual cycles and other individual differences.^12^ Previous research suggests that prediction errors in last menstrual period-based dating are associated with maternal, paternal and fetal characteristics.^15^ Factors such as parity,^15 16^ maternal age,^16^ height and weight^17 18^ and risk behaviours such as smoking^19 20^ have all been linked to variation in prediction accuracy. Yet most existing studies examine these factors individually or in simple multivariable models^21 22^ despite strong correlations and potential interactions among them. As a result, it remains unclear whether incorporating maternal characteristics can improve the joint prediction of delivery timing when such information is otherwise ignored.

The aim of this paper is to assess whether the accuracy of Naegele’s rule-based expected due date prediction can be improved for low-risk pregnancies, where complications are not expected, by using a machine-learning model that incorporates information on maternal characteristics available early in pregnancy. We focus on a context where ultrasound dating is available as a reference standard for model development and evaluation, enabling us to train and evaluate alternative prediction models, but our target use-case is situations where ultrasound-based dating is unavailable or infeasible while the routinely collected information and basic digital infrastructure required to apply the model are available, including early in pregnancy before ultrasound dating has been performed. We do not seek to redefine what constitutes a “normal” gestational duration, nor do we propose changes to clinical thresholds for preterm or post-term delivery or for obstetric interventions. Rather, our contribution is to examine whether a flexible prediction model can reduce the expected due date errors inherent in Naegele’s rule.

Using data on more than 600 000 singleton pregnancies from the Swedish Medical Birth Register, we estimate regression forest models that predict gestational age at delivery based on Naegele’s rule combined with maternal factors such as parity, age, height, weight, nicotine use, hypertension and diabetes. We then compare their predictive performance with that of Naegele’s rule and ultrasound-based expected due date under a common set of inclusion criteria. Our goal is to evaluate whether machine-learning-based methods can provide practical improvements over Naegele’s rule in contexts where ultrasound is not available, without making any claims about replacing ultrasound in clinical practice.

## Materials and methods

### Data source and study population

We use population-wide data from the Swedish Medical Birth Register, which contains detailed information on virtually all births in Sweden since 1973.^23^ The register includes maternal characteristics, pregnancy and delivery information and both Naegele’s rule and ultrasound-based expected due date. We focus on births occurring from 2008 to 2017, a period during which routine ultrasound screening was consistently implemented and conducted according to standardised measurement procedures.^24 25^ This period was selected to provide a relatively consistent clinical benchmark for comparing the dating methods. As a sensitivity analysis, we assess the stability of ultrasound, Naegele’s rule and machine-learning prediction performance across consecutive 2-year birth-period groups (Model evaluation section).

To ensure comparability across dating methods, we apply a series of inclusion criteria commonly used in previous evaluations of expected due date accuracy. These criteria are designed to improve comparability across methods and to define a common prediction target based on spontaneous delivery timing. We restrict the sample to singleton pregnancies with spontaneous onset of labour. Induced deliveries and planned caesarean sections are excluded because their observed delivery dates are directly influenced by clinical management and therefore do not provide the same outcome for evaluating prediction of spontaneous delivery timing. This approach is consistent with previous studies evaluating expected due date accuracy using spontaneous deliveries.^14 22 26^ Moreover, we restrict to pregnancies resulting in newborns surviving at least 28 days. Stillbirths and neonatal deaths were excluded because these outcomes are strongly associated with pathological conditions (eg, fetal growth restriction or placental dysfunction) that can shorten or otherwise alter gestational duration, and including them could bias estimates of prediction accuracy.^19^ We exclude pregnancies lacking information on the reported first day of the last menstrual period, pregnancies without a completed routine ultrasound examination and those missing data on key maternal predictors used in the machine-learning model. To ensure that Naegele’s rule, ultrasound and machine-learning predictions are evaluated within the same term relevant range, we further restrict the sample to pregnancies for which both Naegele’s rule- and ultrasound-based gestational age at delivery fall between 37 and 42 completed weeks, that is, we exclude preterm and post-term deliveries.^27^ This restriction reflects our focus on term pregnancies with spontaneous delivery, consistent with previous studies evaluating expected due date accuracy.^14 22 26^ To assess whether requiring both Naegele’s rule- and ultrasound-based gestational age to fall within the 37–42 week range affects the relative performance of the dating methods, we conduct an additional sensitivity analysis using an expanded evaluation sample, as described in the Model evaluation section.

After all exclusions, the analytic sample consists of 603 861 pregnancies. Appendix A provides a detailed account of the inclusion criteria and case counts at each step.

#### Implications of the inclusion criteria

These restrictions allow for a fair comparison between Naegele’s rule, ultrasound and machine-learning-based dating methods under identical conditions. However, they also limit the generalisability of our findings, as the model has not been trained on data containing preterm and post-term pregnancies, multiple gestations or deliveries initiated by induction or caesarean section, where the determinants of gestational duration may differ and the clinical role of dating accuracy may be more consequential. In particular, restricting the analysis to spontaneous term deliveries may narrow the observed difference between Naegele’s rule and ultrasound, since ultrasound may be relatively more accurate in an unrestricted pregnancy gestational length domain. The model should therefore be interpreted as quantifying improvements in Naegele’s rule-based dating for spontaneous term deliveries, rather than as a comparison of the overall clinical value of Naegele’s rule, machine-learning and ultrasound dating across the full pregnancy population.

Details on missing predictor data and the rationale for complete-case analysis are provided in Predictors section.

### Predictors

The machine-learning model uses information available early in pregnancy and routinely collected in maternal healthcare. We focus on predictors that are consistently recorded at the first antenatal visit, widely available across healthcare settings and reliably measured without specialised diagnostic equipment. This ensures that the model remains feasible and low-cost, and that it can be used in contexts where ultrasound or more detailed clinical measurements are unavailable. All selected variables have been linked in previous research to variation in gestational duration, although estimated associations vary across studies and should not be interpreted causally. These predictors are included strictly for statistical prediction, and their inclusion does not imply that associated differences in gestational age at delivery represent healthy, pathological or desirable biological variation. We include the following predictors:

- Parity: coded as the number of previous births resulting in a liveborn infant, with values capped at 4. Earlier studies report that first-time mothers tend to have longer and more variable gestational duration than women who have given birth earlier.^15 16^
- Maternal age: age in years, at the first antenatal visit. The literature finds mixed patterns, with some studies documenting shorter gestational duration among older mothers and others reporting a U-shaped or inconclusive relationship.^16 21 22 26^
- Maternal height: height in centimetres at the first antenatal visit. Shorter stature has been associated with slightly earlier deliveries, whereas taller mothers tend to have marginally longer gestations on average.^28 29^
- Maternal weight: weight in kilograms at enrolment in antenatal care, prior to pregnancy-related weight gain. Higher maternal weight or body mass index (BMI) has been linked to an increased likelihood of prolonged gestation and post-term delivery.^30 31^
- Nicotine use: indicator equal to one if the mother reported smoking or using snus, a smokeless oral tobacco product common in Sweden, within 3 months prior to pregnancy. Smoking and other nicotine use have consistently been associated with elevated risks of preterm delivery and, on average, shorter gestational duration.^19 20^
- Hypertension: indicator equal to one if the mother reported current or previous chronic hypertension. Chronic hypertensive disorders are associated with a higher probability of medically indicated early delivery and a general tendency toward shorter gestation.^32^
- Diabetes: indicator equal to one if the mother reported current or previous diabetes mellitus (type I or II; gestational diabetes is not included). Pre-existing diabetes has been linked to increased risks of early delivery, partly due to related medical complications.^33^

Summary statistics for all predictors are provided in online supplemental appendix B.

#### Handling of missing data

A small share of pregnancies (3.1%) lacked information on one or more maternal predictors, primarily height or weight recorded at the first antenatal visit. We chose complete-case analysis rather than multiple imputation for two reasons. First, missingness in these variables reflects incomplete antenatal registration rather than underlying maternal characteristics, making imputation heavily assumption-dependent. Second, the prediction model is intended for settings where these routinely collected measurements are available at the first visit; restricting the analytic sample to pregnancies with complete early-pregnancy records, therefore, aligns with the intended use case.

### Outcome definition

The outcome of interest is the actual date of delivery, expressed as gestational age at delivery (in days since last menstrual period). Prediction performance is evaluated by comparing predicted and observed delivery dates. For consistency with prior work and clinical relevance, we assess accuracy using four complementary metrics:

- Exact-day accuracy: the proportion of predictions matching the observed delivery date exactly.
- ±1 day accuracy: the proportion of predictions within 1 day of the observed date.
- ±7 day accuracy: the proportion of predictions within 1 week of the observed date.
- Mean squared error (MSE) and root mean squared error (RMSE): summary measures of the distribution of prediction errors.

### Dating methods compared

We compare three approaches for estimating the expected due date:

- Naegele’s rule: expected due date=recorded first day of the last menstrual period+280 days.
- Ultrasound-based dating: expected due date is calculated by projecting the gestational age estimated at the routine second trimester ultrasound scan forward to 280 days. Gestational age at the scan is derived from established biometric reference charts (eg, crown–rump length, biparietal diameter, head circumference).^4 28 34 35^ This is the clinical reference standard in Sweden during the study period.
- Machine-learning model: predicted date of delivery obtained from the regression-forest model described in Machine-learning model section.

All three methods are evaluated in the same test dataset and under identical inclusion criteria.

### Machine-learning model

We use a regression-forest model to predict gestational duration based on Naegele’s rule and the maternal predictors described in Predictors section. Regression forests offer a flexible, non-parametric approach that accommodates nonlinear relationships and interactions without imposing strong functional-form assumptions.^36^ This flexibility is useful in the present setting, where relationships between maternal characteristics and gestational duration may be nonlinear or interactive, while the model also allows straightforward computation of variable-importance measures.^37^ We did not undertake a systematic comparison across model classes and therefore do not claim that the regression forest is the optimal estimator for this prediction problem.

The model is implemented using the grf package in *R*, following the generalised random forest framework.^36 38^ Each forest consists of an ensemble of decision trees, where splits are chosen from randomly selected subsets of predictors. This approach reduces overfitting and improves out-of-sample performance. Final predictions are obtained by averaging predictions across all trees in the forest.

A simplified illustration of a decision tree is provided in online supplemental figure S1 to demonstrate how combinations of maternal characteristics define partitions of the predictors.

Because prediction from a regression forest reduces to averaging terminal-node means across trees, the fitted model can be represented exactly as a set of split rules and terminal-node means. Producing a prediction, therefore, requires only a tree traversal and an average, and no specialised computing infrastructure.

### Model implementation

No formal sample-size calculation was performed. The study size was determined by the number of eligible pregnancies available in the Swedish Medical Birth Register during the prespecified study period. Methodological guidance for prediction-model development emphasises the importance of having a sufficiently large sample relative to the number of candidate predictors to limit overfitting and obtain precise estimates of predictive performance.^39 40^ Previous studies applying machine-learning methods to gestational-age prediction have generally been based on substantially smaller samples.^41–45^ Given the large available sample (n=6 03 861), the limited number of candidate predictors and the use of separate training, validation and independent test samples, the available sample was considered sufficient for model development and evaluation.

We follow a structured process for model training and evaluation. The full dataset is randomly partitioned into three disjoint subsets using a fixed random seed: a training set (60%), a validation set (20%) and a test set (20%). The training set is used to estimate candidate models, the validation set is used for hyperparameter selection, and all final performance metrics are computed exclusively on the independent test set to ensure an unbiased evaluation.

Candidate models are trained on the training data, and their predictive accuracy is assessed on the validation data. The hyperparameter combination that minimises the MSE in the validation data is selected as the optimal configuration. The selected model is then applied to the test data, and its accuracy is compared with that of Naegele’s rule and ultrasound under identical inclusion criteria using the performance metrics described in Outcome definition section.

### Hyperparameter tuning

The grf package allows several hyperparameters to be tuned to balance model flexibility and predictive stability. We use a grid-search procedure to identify the combination that yields the lowest validation-set MSE.^46 47^ The hyperparameters considered include: the number of trees in each forest (num.trees); the fraction of the training data used to construct each tree (sample.fraction); the number of predictors randomly selected for splitting at each node (mtry); the minimum variance reduction required for a split (alpha); and the minimum number of observations required in a terminal node (min.node.size).

The full grid of candidate values and the final selected configuration are reported in online supplemental appendix C.

### Model evaluation

After selecting the best-performing model—that is, the regression forest with the hyperparameter combination yielding the lowest MSE in the validation data—we evaluated predictive accuracy using the independent test dataset. All performance metrics for the primary analysis were computed exclusively on the test set to ensure an unbiased assessment of model performance.

We summarise accuracy using the metrics described in Outcome definition section: exact-day accuracy, ±1 day accuracy, ±7 day accuracy, MSE and RMSE. To formally compare predictive performance between methods, we additionally calculated paired differences in each performance metric within the same independent test set. CIs and p values for these differences were obtained using paired non-parametric bootstrap resampling with 1000 replications. For accuracy measures, positive differences indicate higher accuracy for the first method; for MSE and RMSE, negative differences indicate lower prediction error. To enable comparison with existing approaches, we evaluated Naegele’s rule-based and ultrasound-based expected due date in the same test dataset and under identical inclusion criteria. Because ultrasound-based dating serves as the clinical reference standard, we quantify the improvement of the machine-learning model relative to Naegele’s rule by reporting the share of the accuracy gap between Naegele’s rule and ultrasound that is closed by the machine-learning model. We refer to this comparative metric as *Gap Closure*. If the machine-learning model performs identically to Naegele’s rule, Gap Closure is 0%; if it matches the accuracy of ultrasound, Gap Closure is 100%. Values above 100% indicate that the machine-learning model outperforms ultrasound on the corresponding accuracy metric.

We also assessed calibration for the machine-learning model by grouping predictions according to the predicted gestational age at delivery (in days) and plotting the observed mean gestational age at delivery within each day-level bin. The resulting calibration curve (online supplemental figure S3) was compared with the 45 degree line corresponding to perfect calibration. Because Naegele’s rule and ultrasound both imply a constant expected gestational duration of 280 days at delivery, they do not yield meaningful calibration curves under a binned evaluation; their calibration reduces to a single point and is therefore summarised numerically via mean prediction errors rather than graphically.

Finally, to aid interpretability and guide future model development, we examined the relative importance of each predictor using the standard split-frequency measure from the regression forest. While variable importance scores do not directly measure the effect of a predictor on accuracy, they describe how frequently each predictor is used to create splits in the trees.

We additionally assessed heterogeneity in model performance and the sensitivity of our results to the primary gestational-age restriction.

First, to assess whether predictive performance was stable across the study period and across the country, we evaluated the primary model separately within strata of the independent test set defined by 2-year birth periods (2008–2009, 2010–2011, 2012–2013, 2014–2015 and 2016–2017) and by geographical region. The same fitted model was applied in every stratum; no retraining or recalibration was performed. For each stratum, we report exact-day, ±1 day and ±7 day accuracy and RMSE for Naegele’s rule, ultrasound and machine-learning, with 95% CIs obtained by non-parametric bootstrap within the stratum. Because all strata were represented in the development data, these analyses assess the stability of performance rather than constituting external validation.

Second, to assess whether the restriction requiring both Naegele’s rule- and ultrasound-based gestational age at delivery to fall between 37 and 42 completed weeks influenced the relative performance of the dating methods in an unrestricted gestational length domain, we conducted a sensitivity analysis using an expanded evaluation sample. The original test dataset was supplemented with a random 20% sample of otherwise eligible spontaneous singleton pregnancies that had been excluded from the primary analytical sample solely because Naegele’s rule-based gestational age, ultrasound-based gestational age or both fell outside the 37–42 week range. The previously trained machine-learning model was applied to these additional observations without retraining or hyperparameter retuning. We evaluated Naegele’s rule, ultrasound and machine-learning using the same performance metrics as in the primary analysis. Because the machine-learning model was developed using pregnancies satisfying the 37–42 week restriction, its performance on observations outside this range was interpreted as a sensitivity analysis of the primary comparative results to the gestational-age restriction.

We further assessed whether predictive performance differed across maternal subgroups and whether subgroup-specific model fitting improved prediction. Separate regression-forest models were estimated for subgroups defined by parity, maternal age, BMI category and nicotine use. Within each subgroup, performance of the subgroup-specific model was compared with Naegele’s rule, ultrasound and predictions from the primary machine-learning model applied to the same observations.

We also examined whether pregnancy history could improve prediction among women with a previous birth. For second births, we estimated an additional regression-forest model that incorporated gestational duration at delivery in the previous pregnancy as an additional predictor alongside the predictors used in the primary model. Predictive performance was evaluated in the corresponding subgroup of the independent test set and compared with Naegele’s rule, ultrasound and predictions from the primary machine-learning model applied to the same pregnancies.

For both sets of analyses, paired differences in performance were evaluated using the bootstrap procedure described above.

### Data availability statement

The study relies on sensitive individual-level data from the Swedish Medical Birth Register, held by the Swedish National Board of Health and Welfare. Ethical and legal restrictions prevent us from sharing these data. Information on how to request access is available at https://bestalladata.socialstyrelsen.se/data-for-forskning/.

All data-processing and analysis code is available as supplementary material with this article. Together with the model specification, hyperparameter settings and random seed reported in the manuscript and online supplemental appendix C, this allows any researcher with authorised access to the register to reproduce the models and all reported results.

The original fitted grf object cannot be publicly shared because it retains individual-level information from the underlying register data. A shareable representation of the fitted forest, containing the split variables, split thresholds and terminal-node predictions but not the underlying individual-level register data, is available from the corresponding author on reasonable request, at no cost, under a written agreement restricting its use to external validation or methodological evaluation. Predictions generated from this representation reproduce those of the fitted model to numerical precision. We do not distribute the model as a ready-to-use tool, because it has not been externally validated and should not be used clinically in its present form. Broader distribution and implementation will be considered only after appropriate external validation, with the aim of ensuring that, where implementation is appropriate, access does not constitute a barrier to use in resource-constrained settings.

### Patient and public involvement statement

Patients and/or the public were not involved in the design, conduct, reporting or dissemination plans of this research.

### Reporting guidelines

This study is reported in accordance with the TRIPOD+AI reporting guideline.

## Results

This section presents the predictive performance of the machine-learning model and compares it with Naegele’s rule and ultrasound-based expected due date, using the common test dataset and identical inclusion criteria. The machine-learning model uses the reported first day of the last menstrual period, as in Naegele’s rule, together with maternal characteristics including parity, age, height, weight, nicotine use, hypertension and diabetes.

### Main performance comparison

Table 1 presents the predictive accuracy of the machine-learning model, Naegele’s rule and ultrasound across five evaluation metrics: exact-day accuracy, accuracy within ±1 and ±7 days of delivery, MSE and RMSE.

**Table 1:** Performance of machine-learning model, Naegele’s rule and ultrasound in predicting the date of delivery (test dataset)

	Exact day	±1 day	±7 days	MSE	RMSE
Machine-learning model	4.90%(4.79 to 5.03)	14.69%(14.50 to 14.89)	65.70%(65.44 to 65.99)	57.78(57.41 to 58.24)	7.60(7.58 to 7.63)
Naegele’s rule	4.75%(4.63 to 4.87)	14.14%(13.95 to 14.35)	63.86%(63.58 to 64.13)	59.00(58.60 to 59.38)	7.68(7.66 to 7.71)
Ultrasound	5.35%(5.22 to 5.48)	15.63%(15.42 to 15.83)	67.92%(67.65 to 68.18)	54.73(53.59 to 56.54)	7.40(7.32 to 7.52)
Gap closure	25.48%	37.06%	45.44%	26.98%	26.61%

Exact-day, ±1 day, and ±7 day accuracy report the percentage of deliveries occurring within the specified interval around the predicted date of delivery. MSE denotes mean squared error and RMSE root mean squared error, both calculated from the difference between predicted and observed gestational duration. Values in brackets are 95% CIs. Gap closure indicates the percentage of the performance gap between Naegele’s rule and ultrasound closed by the respective machine-learning model; values above 100% indicate performance exceeding ultrasound on that metric. Data source: Swedish Medical Birth Register (*Medicinska födelseregistret*), 2008–2017.

MSE, mean squared error; RMSE, root mean squared error.

Descriptive distributions of actual and predicted delivery timing are reported in online supplemental table S3. Expressed as delivery timing relative to last menstrual period, actual delivery occurred at a mean of 280.90 days (median 282; IQR 276–287), compared with 280 days by definition for Naegele’s rule, 280.75 days for the ultrasound-predicted due date (median 280; IQR 278–284) and 280.90 days for the machine-learning prediction (median 280.96; IQR 280.51–281.35).

The machine-learning model modestly improves on Naegele’s rule-based dating across all accuracy metrics. Exact-day accuracy increases from 4.75% (Naegele’s rule) to 4.90% (Model), and accuracy within ±7 days increases from 63.86% to 65.70%. MSE is reduced from 59.00 to 57.78. Paired bootstrap comparisons confirmed improvements in all five performance metrics (online supplemental table S4). For the primary metric, accuracy within ±7 days was 1.85 percentage points higher for the model than for Naegele’s rule (95% CI 1.72 to 2.00), corresponding to approximately one additional correct prediction within ±7 days per 54 pregnancies. The corresponding improvements were 0.55 percentage points for ±1 day accuracy (95% CI 0.38 to 0.72; approximately one additional correct prediction per 182 pregnancies) and 0.15 percentage points for exact-day accuracy (95% CI -0.01 to 0.32; approximately one additional correct prediction per 667 pregnancies). MSE and RMSE were also lower for the model than for Naegele’s rule. Although these improvements are small in absolute terms, they close between 25% and 45% of the accuracy gap between Naegele’s rule and ultrasound, depending on the metric. Ultrasound remains more accurate than the model across all five performance metrics, with paired comparisons favouring ultrasound (online supplemental table S4). Calibration of the machine-learning model is shown in online supplemental figure S3. When predictions are grouped by predicted gestational age at delivery (in days), the observed mean gestational age at delivery in each bin lies close to the 45-degree line across the model’s prediction range. Deviations are larger in the sparsely populated extreme bins. Overall, this indicates little systematic bias across the range in which most predictions occur.

### Relative importance of predictors

To aid interpretability, we assessed the relative importance of predictors using the standard split-frequency measure from the regression forest. Maternal weight, height and age appear most frequently in decision splits, followed by parity and nicotine use, whereas hypertension and diabetes contribute little to partitioning. These importance scores provide descriptive insight into how the model organises the predictor space, but they should not be interpreted as causal effects. A full variable-importance plot is provided in online supplemental figure S2).

### Stability and sensitivity analyses

Prediction performance was broadly stable across the study period (online supplemental figure S5). Ultrasound remained the most accurate method in each 2-year birth-period group for exact-day, ±1 day and ±7 day accuracy. Although some variation was observed across periods, there was no indication of a systematic improvement in ultrasound prediction accuracy over 2008–2017. The machine-learning model likewise consistently outperformed Naegele’s rule throughout the study period. Performance was also similar across geographical regions (online supplemental figure S6), with ultrasound outperforming both alternative methods and the model outperforming Naegele’s rule in Götaland, Svealand and Norrland across all three accuracy measures.

We additionally examined the sensitivity of the results to the primary restriction requiring both Naegele’s rule- and ultrasound-based gestational age at delivery to fall between 37 and 42 completed weeks. For this analysis, the previously trained model was evaluated in an expanded evaluation sample that included a random 20% sample of otherwise eligible pregnancies excluded solely because Naegele’s rule-based gestational age, ultrasound-based gestational age or both fell outside this range. The model was applied without retraining or recalibration. In the expanded evaluation sample, accuracy within ±7 days was 56.94% for the model, compared with 55.28% for Naegele’s rule and 61.82% for ultrasound. Thus, the model continued to outperform Naegele’s rule, while the advantage of ultrasound over both Naegele’s rule and the model was larger than in the primary analysis. For ±7 day accuracy, the proportion of the Naegele’s rule–ultrasound gap closed by the machine-learning model decreased from 45.44% in the primary analysis to 25.27% in the expanded evaluation sample. These results indicate that the 37–42 week restriction attenuated the observed performance difference between ultrasound and the other dating methods, while the improvement of the model over Naegele’s rule persisted. Full results are reported in online supplemental table S5.

### Subgroup analyses

We next examined whether predictive performance improved when separate machine-learning models were trained for subgroups defined by parity, maternal age, BMI and nicotine use. Across subgroups, both the primary and subgroup-specific machine-learning models generally improved prediction relative to Naegele’s rule but remained less accurate than ultrasound (online supplemental ftable S6 and S6b). Direct paired comparisons showed that subgroup-specific model fitting provided no meaningful additional improvements over the primary machine-learning model. Although some differences were statistically significant, their magnitudes were generally modest and varied across subgroups and performance metrics. Thus, tailoring the machine-learning model to these maternal subgroups did not materially alter predictive performance.

### Can information from the first birth help predict the second?

Finally, among second time mothers, we examined whether incorporating gestational duration from the previous pregnancy improved prediction of delivery timing. The second-birth model performed better than both Naegele’s rule and the primary machine-learning model applied to the same pregnancies (table 2 and online supplemental table S4a). Accuracy within±7 days was 70.48% for the second-birth model, compared with 65.25% for Naegele’s rule, 67.34% for the primary machine-learning model and 69.64% for ultrasound. In paired comparisons, the second-birth model increased±7 day accuracy by 5.23 percentage points relative to Naegele’s rule (95% CI 4.86 to 5.57; p<0.001) and by 3.14 percentage points relative to the primary machine-learning model (95% CI 2.79 to 3.45; p<0.001). Compared with ultrasound, the second-birth model increased±7 day accuracy by 0.84 percentage points (95% CI 0.34 to 1.31; p<0.001) and had lower MSE and RMSE, whereas exact-day accuracy did not differ significantly.

**Table 2:** Predictive performance among second births in the independent test set (n=40 781)

	Exact day	±1 day	±7 days	MSE	RMSE
Machine-learning model	4.99%(4.78 to 5.21)	15.18%(14.83 to 15.51)	67.34%(66.92 to 67.79)	54.24(53.69 to 55.01)	7.36(7.33 to 7.42)
Machine-learning model (second birth)	5.48%(5.27 to 5.71)	16.47%(16.15 to 16.83)	70.48%(70.05 to 70.95)	49.95(49.26 to 50.57)	7.07(7.02 to 7.11)
Naegele’s rule	4.91%(4.68 to 5.12)	14.61%(14.28 to 14.93)	65.25%(64.81 to 65.67)	56.27(55.63 to 56.91)	7.50(7.46 to 7.54)
Ultrasound	5.43%(5.21 to 5.66)	15.98%(15.62 to 16.35)	69.64%(69.17 to 70.11)	50.67(50.00 to 51.32)	7.11(7.07 to 7.16)
Machine-learning model Gap Closure	15.09%	41.29%	47.62%	36.19%	35.59%
Machine-learning model (second birth) Gap Closure	109.43%	135.73%	119.23%	112.74%	113.13%

Estimates are based on second births in the independent test set (n=40 781). Machine-learning model denotes predictions from the main machine-learning model using maternal characteristics available for all pregnancies. Machine-learning model (second birth) denotes predictions from the second-birth machine-learning model, which additionally incorporates gestational duration of the previous pregnancy. Exact-day, ±1 day and ±7 day accuracy report the percentage of deliveries occurring within the specified interval around the predicted date of delivery. MSE denotes mean squared error and RMSE root mean squared error, both calculated from the difference between predicted and observed gestational duration. Values in brackets are 95% CIs. Gap closure indicates the percentage of the performance gap between Naegele’s rule and ultrasound closed by the respective machine-learning model; values above 100% indicate performance exceeding ultrasound on that metric. Data source: Swedish Medical Birth Register (*Medicinska födelseregistret*), 2008–2017.

MSE, mean squared error.; RMSE, root mean squared error.

These results indicate that gestational duration from the previous pregnancy contains predictive information beyond the routinely collected maternal characteristics included in the primary model. However, this analysis is restricted to second births with available information from the previous pregnancy and therefore does not generalise to first births or to pregnancies without reliable longitudinal information. A variable-importance plot for the second-birth model is provided in online supplemental figure S4.

## Discussion

This study examined whether the accuracy of expected due date, calculated using Naegele’s rule, can be improved by incorporating routinely recorded maternal characteristics into a machine-learning prediction model. Using data on more than 600 000 singleton pregnancies from the Swedish Medical Birth Register, we developed and evaluated regression-forest models that predict gestational duration under the same inclusion criteria applied to Naegele’s rule and ultrasound dating. Across all accuracy metrics, the machine-learning model provided consistent improvements over Naegele’s rule, reducing between one-quarter and nearly one-half of the accuracy gap between Naegele’s rule and ultrasound, while ultrasound remained more accurate overall. In settings where ultrasound is unavailable but the information and basic digital infrastructure required to apply the model are available, such improvements may have practical value for planning and early pregnancy management.

Although the improvement over Naegele’s rule was statistically robust, its magnitude in the full sample was modest. For the primary performance metric, machine-learning increased accuracy within±7 days by 1.87 percentage points (95% CI 1.72 to 2.01), corresponding to approximately one additional prediction within 1 week per 54 pregnancies. There is no established minimum clinically important difference for expected due date prediction, and predictive accuracy alone does not establish clinical benefit. The practical importance of this improvement therefore depends on the setting and intended use and ultimately requires evaluation of its consequences for clinical decision-making and perinatal outcomes.

The predictors contributing most strongly to the machine-learning model included maternal weight, height and age, followed by parity and nicotine use. These patterns are broadly consistent with earlier research documenting associations between maternal characteristics and variation in delivery timing. Our model captures these relationships jointly and non-linearly, without imposing parametric assumptions, offering a more flexible approach than previously used multivariable models.

Training separate models for subgroups defined by parity, maternal age, BMI and nicotine use yielded only small additional improvements over the primary machine-learning model. Although some differences were statistically significant, the gains were generally modest and inconsistent across performance metrics. This suggests that subgroup-specific model fitting based on these routinely observed maternal characteristics adds limited predictive value beyond that captured by the primary model. A larger improvement was observed for second births when gestational duration from the previous pregnancy was added as a predictor. The second-birth model clearly outperformed both Naegele’s rule and the primary machine-learning model applied to the same pregnancies. Compared with ultrasound, it achieved modestly higher±7 day accuracy and lower prediction error, while exact-day accuracy was similar. These findings indicate that gestational duration in a previous pregnancy contains predictive information beyond the routinely collected maternal characteristics included in the primary model. However, this approach is applicable only when information from a previous pregnancy is available and therefore cannot be used for first pregnancies.

Several limitations should be noted. First, the primary analysis is restricted to spontaneous singleton births between 37 and 42 weeks of gestation. While this restriction ensures comparability across dating methods, it is not trained on preterm and post-term deliveries, where dating accuracy may be more clinically consequential. However, our sensitivity analysis further indicated that the machine-learning model continued to outperform Naegele’s rule when pregnancies outside the primary gestational-age range were included. Second, all predictors aside from last menstrual period were derived from routine early pregnancy records; we did not incorporate fetal biometry or biomarkers, which are central to ultrasound-based dating. Third, the model was developed and evaluated within a single national register covering a single decade. Internal validation was strict, and performance was stable across birth periods and regions, but all evaluation remains internal: the model has not been tested in data generated outside the Swedish Medical Birth Register. Predictive performance may differ in populations with different demographic profiles, different distributions of the included predictors or lower accuracy in the reporting of the last menstrual period. Because the model was developed using complete cases, its performance when predictor values are missing or measured with substantial error has not been established. The model should therefore not be applied when required predictor information is unavailable. External validation should assess the reliability of predictor measurement and, where necessary, recalibration before the model is used for decision support in another setting. Fourth, we selected a regression forest for its ability to accommodate nonlinearities and interactions, but we did not systematically compare alternative model classes. Other estimators could yield different predictive performance using the same predictors, and our results should therefore not be interpreted as identifying the optimal prediction model. The model also targets a point prediction by minimising squared error; alternative prediction targets, such as prediction intervals or probabilities of delivery within clinically relevant windows, may be more appropriate for some clinical applications. Finally, this study evaluates predictive accuracy rather than the clinical consequences of using machine-learning-based expected due date predictions. Although the observed improvements over Naegele’s rule may be relevant where ultrasound is unavailable, our analysis does not establish that using these predictions improves clinical decision-making or perinatal outcomes. The resources required to develop the model differ substantially from those required to apply a previously developed model. Development required a large population register and substantial computing capacity, whereas generating a prediction from the fitted model requires only a sequence of threshold comparisons and aggregation across trees. If externally validated, the model could therefore, in principle, be implemented in a lightweight digital application without requiring the computational infrastructure used for model development. Users would need to enter or retrieve the required maternal characteristics and last menstrual period information, but calculation of the prediction itself could be automated and would not require specialist machine-learning expertise. Nevertheless, such implementation would still require access to basic digital infrastructure and reliable measurement of the required predictors. The potential use case should therefore be understood as settings where ultrasound dating is unavailable but the information and digital resources required to apply the model are available, rather than low-resource settings generally.

The use of machine-learning models for expected due date prediction also raises ethical and methodological considerations regarding interpretability, overfitting and differential performance across population groups. A regression forest is less transparent than Naegele’s rule, and variable-importance measures describe the model globally rather than explaining individual predictions. We therefore report variable importance for the primary and second-pregnancy models (online supplemental figures S2 and S4) and calibration of the primary model (online supplemental figure S3) and provide the model specification, hyperparameter settings, and analytical code to facilitate reproducibility and independent evaluation. To limit overfitting, hyperparameters were selected exclusively in a separate validation sample and final performance was evaluated in an independent test sample. Performance was also assessed across birth periods and geographical regions without retraining or recalibration. Nevertheless, calibration showed larger deviations in sparsely populated parts of the prediction range, and improvements over Naegele’s rule were not uniform across maternal subgroups. In particular, the model did not consistently improve on Naegele’s rule among women with BMI at or below 18.5 (online supplemental table S6). These findings underscore the importance of assessing calibration and subgroup performance when evaluating the model in other populations.

Any clinical application would also require careful consideration of how predictions are communicated and used. An individualised predicted date should not be interpreted as certainty about the timing of delivery, and the model should be regarded as a potential decision-support tool for planning and counselling rather than as a standalone rule for clinical intervention. In particular, predictions should not be used mechanically to determine interventions such as induction or intensified monitoring. Clinical implementation would require external validation, assessment of calibration and subgroup performance in the target population, and prospective evaluation of potential effects on perinatal outcomes, with clinical decisions remaining the responsibility of healthcare professionals. The fitted model is therefore not currently distributed as a ready-to-use clinical tool, and broader clinical implementation should await external validation and evaluation in the intended target populations.

With these limitations and considerations in mind, our findings show that incorporating maternal characteristics into flexible prediction models can reduce dating errors inherent in Naegele’s rule. This approach may provide useful decision support in settings where ultrasound dating is unavailable but the required maternal information and basic digital infrastructure are available. More broadly, the results illustrate how combining population data with modern prediction tools can produce individualised predictions of delivery timing. Future research could extend these findings by incorporating additional predictors—such as menstrual cycle characteristics, biomarkers, or environmental exposures—and by validating machine-learning-based models in populations with different demographic and clinical profiles. Another promising direction is to combine maternal characteristics with simplified ultrasound or biometric measurements, which shows potential for substantial improvement in gestational age estimation.^41–45^ Further work is also needed to evaluate how prediction models can be integrated into clinical practice and planning, and whether improved expected due date estimates can meaningfully influence obstetric decision-making in environments without routine access to ultrasound. Future studies could additionally compare alternative model classes and prediction targets, including approaches that quantify individual prediction uncertainty or estimate the probability of delivery within clinically relevant time windows.

In sum, incorporating routinely collected maternal characteristics into a regression-forest model provided a statistically robust but modest improvement in due-date prediction compared with Naegele’s rule among spontaneous term pregnancies. In the full sample, the improvement corresponds to approximately one additional prediction within±7 days per 54 pregnancies, while ultrasound remains more accurate overall. Whether improvements of this magnitude are clinically meaningful will depend on the setting and intended use and cannot be determined from predictive accuracy alone. The model should therefore not be regarded as a substitute for ultrasound-based dating, and external validation and evaluation of clinical consequences are required before applied use.

## Supplementary material

10.1136/bmjopen-2026-120970 online supplemental file 1

## Data Availability

Data may be obtained from a third party and are not publicly available.
