# Supplementary material for: Can maternal characteristics improve due date prediction without ultrasound? Development and internal validation of a machine-learning prediction model in 600 000 pregnancies in Sweden
Source: BMJ Open. 2026 Oct 23;16(10):e120970. doi: 10.1136/bmjopen-2026-120970 (PMC13711609; doi:10.1136/bmjopen-2026-120970)
Supplement: Supplementary file 1 [file bmjopen-2026-120970.reviewer_comments.pdf]

PEER REVIEW HISTORY

BMJ Open publishes all reviews undertaken for accepted manuscripts. Reviewers are asked to complete a checklist review form and are provided with free text boxes to elaborate on their assessment. These free text comments are reproduced below.

ARTICLE DETAILS

Title (Provisional)

Can maternal characteristics improve due date prediction without ultrasound? Development and internal validation of a machine-learning prediction model in 600,000 pregnancies in Sweden

Authors

Gilson, Dutra; Elinder, Mikael; Erixson, Oscar

VERSION 1 - REVIEW

**Reviewer**	**1**
**Name**	**Pripp, Are**
**Affiliation**	**Oslo University Hospital, Department of Biostatistics, Epidemiology & Health Economy**
**Date**	**18-Jun-2026**
**COI**	**None**

The paper should be of interest for readers of the journal. I recommend it for publication. Would it be possible to externally validate the ML model in a new external dataset?

**Reviewer**	**2**
**Name**	**Sande, Ragnar**
**Affiliation**	**Stavanger University Hospital, Department of Obstetrics and Gynaecology**
**Date**	**21-Jun-2026**
**COI**	**None**

This is a well-written paper that should be of interest to the readers of BMJ Open. I have a few issues that I would like to see addressed:

1. The paper states only the difference in predicted and actual delivery dates; I would very much like to see the precise gestational age for predicted and actual delivery. If nothing else a table stating mean with 95% confidence intervals and median with interquartile ranges for due date by ultrasound, Naegele, machine learning and actual delivery.

2. The limitations paragraph in the discussion section should address the ethical aspects of machine learning in medical research, including black box concerns and the risks of overfitting.

3. I would like to see the model used as supplementary material with the paper. The paper states that the presented model may be of use in low-resource settings, so providing this model cost free to the intended users would be prudent. This would also allay some of the questions arising from the conflicting interest statement.

The paper is well written and in good English, I have however spotted two typos:

Page 5 line 10 duplicate

Page 7 line 28 incomplete sentence

**Reviewer**	**3**
**Name**	**Cai, Guoqi**
**Affiliation**	**Anhui Medical University School of Public Health**
**Date**	**07-Jul-2026**
**COI**	**None**

Gilson and colleagues conducted an interesting and potentially important study evaluating whether ML can improve the predictive accuracy of due date compared to Naegele's rule, and whether it might even replace ultrasound-based dating (especially in places without such devices). The manuscript reads well and sufficient details are provided. However, I have some concerns which are shown below.

1. The population is highly selected, enrolling delivery between 37 and 42 weeks. This may have overlooked the beneficial effect of US as the date of delivery is influenced, if not determined, by the findings of US (especially at later stages of pregnancy), making the accuracy of NR and US similar and the minor improvement of ML models compared to NR.

2. Another important issue is that the small discrepancies of accuracies are neither compared statistically nor discussed clinically.

3. The conclusion may change entirely if ML does not provide significant improvement in accuracy of delivery date, statistically or clinically, compared to NR.

**Reviewer**	**4**
**Name**	**Zewotir, Temesgen**
**Affiliation**	**University of KwaZulu-Natal, Statistics**
**Date**	**07-Jul-2026**
**COI**	**None**

This study evaluates whether machine-learning (ML) methods can improve gestational age estimation over Naegele’s Rule (NR) in contexts where ultrasound (US) is unavailable. The authors emphasize that this method is intended to complement early pregnancy care, not to replace ultrasound in clinical practice.The Swedish Medical Birth Register, analyzing over 600,000 singleton pregnancies between 2008 and 2017. This period reflects a timeframe where routine ultrasound screening was standardized and consistently

implemented.Regression forest models were trained using the reported first day of the last menstrual period (LMP) combined with maternal characteristics.Parity, age, height, weight, nicotine use, hypertension, and diabetes.The predictive performance of the ML model was compared directly against both Naegele's Rule (NR) and ultrasound-based estimated due dates (EDD) using a common test dataset and identical inclusion criteria.The ML model closed between one-quarter and nearly one-half of the accuracy gap that typically exists between NR and ultrasound dating.

1. Ultrasound predictions inherently rely on diverse statistical and machine-learning models (such as multivariable linear and quantile regressions) to calculate biometric estimates. Because ultrasound accuracy has evolved over time, the study would be more significant if it analyzed how historical improvements in US technology compare to this new ML model. Without this baseline, the exact scientific contribution of the study is difficult to measure.

2. The study relies entirely on a single dataset from a specific geographic and demographic population (Sweden). A universal conclusion cannot be drawn from a single data source, as it lacks external validation. Furthermore, the ML model's accuracy may have been artificially limited by the software used; utilizing a general-purpose prediction software may have yielded lower/higher accuracy than a specialized, medically optimized algorithm.

3. Because ultrasound dating is not 100% perfect, a more valuable research objective for the machine learning model would have been investigating how incorporating maternal characteristics directly into ultrasound data could improve overall US accuracy. Enhancing ultrasound precision represents a more meaningful clinical advancement than simply optimizing the less reliable Naegele's Rule.

4. The authors argue that this model is highly useful in environments where ultrasound technology is unavailable. However, the proposed regression forest model is a complex, computer-intensive algorithm that cannot be performed manually. In short, the model requires advanced digital infrastructure to run, yet it yields less accurate results than an ultrasound. This creates a practical paradox, making it unusable in the very low-resource areas it claims to benefit

---

VERSION 1 - AUTHOR RESPONSE

Reviewer 1

Comment 1:

The paper should be of interest for readers of the journal. I recommend it for publication. Would it be possible to externally validate the ML model in a new external dataset?

Response 1:

We thank the reviewer for the positive assessment and for raising external validation, which we agree is an essential next step before any applied use of the model.

We have addressed this point in three ways.

First, we have clarified the study type throughout the manuscript. Following TRIPOD+AI, the paper is now explicitly described as reporting the development and internal validation of a prediction model. This is stated in the Title, Abstract, Methods, and Discussion so that the results are not interpreted as describing an externally validated model ready for clinical use.

Second, we have strengthened the assessment of model performance within the available data. Model development and internal validation used a strict train–validation–test partition, with hyperparameters selected exclusively in the validation sample and final performance evaluated in an independent test set of 120,771 pregnancies that was not used for model fitting or hyperparameter selection. We have additionally evaluated the fitted model across consecutive two-year birth periods and geographical regions within the independent test set (new Supplementary Figures S5 and S6, discussed in new Section 3.3). Performance was broadly stable across these strata, with US remaining the most accurate method and ML consistently outperforming NR. We also conducted a sensitivity analysis using an expanded evaluation sample that included a random 20% sample of otherwise eligible spontaneous singleton pregnancies excluded from the primary analysis because NR-based gestational age, US-based gestational age, or both fell outside the 37–42-week range (new Supplementary Table S5, Section 3.3).

None of these additional analyses constitutes proper external validation. The temporal and geographical strata originate from the same national register and were represented in the development data, while the expanded-sample analysis applies a model developed in the term-pregnancy sample to observations outside that restriction. These analyses therefore assess the stability and sensitivity of the internally validated results but do not establish transportability to an independent population.

Third, we have narrowed the conclusions accordingly. The revised limitations section explicitly states that the model was developed and evaluated within a single national register and has not been tested in data generated outside the Swedish Medical Birth Register. We therefore state that external validation, and potentially recalibration, are required before use in another population and have removed wording that could imply that the model is ready for clinical deployment.

External validation in a genuinely independent dataset is not possible with the data currently available to us and therefore lies outside the scope of the present study. We agree with the reviewer that this is the appropriate next step. We have identified external validation in populations with different demographic and clinical characteristics as a priority for future research. To facilitate such evaluation, all data-processing and analysis code will be made publicly available, and the fitted model will be available to researchers for external validation or methodological evaluation as described in the Data Availability statement.

Reviewer 2

Comment 1:

The paper states only the difference in predicted and actual delivery dates; I would very much like to see the precise gestational age for predicted and actual delivery. If nothing else a table stating mean with 95% confidence intervals and median with interquartile ranges for due date by ultrasound, Naegele, machine learning and actual delivery.

Response 1:

We agree and have added a new supplementary table (Table S3) based on the independent test set (N=120,771), reporting means with 95% confidence intervals and medians with interquartile ranges for actual delivery timing and delivery timing predicted by NR, US, and ML, which referred to in Section 3.1.

To ensure that actual and predicted delivery timing are directly comparable, all quantities are expressed as days from the reported first day of the last menstrual period (LMP).

Comment 2:

The limitations paragraph in the discussion section should address the ethical aspects of machine learning in medical research, including black box concerns and the risks of overfitting.

Response 2:

We agree and have expanded the Discussion to address ethical and methodological considerations associated with the use of machine learning for clinical prediction, including interpretability, overfitting, differential performance across subgroups, and appropriate conditions of use. These additions also address relevant transparency and fairness considerations in the TRIPOD+AI reporting framework, which we now follow at the editor's request.

Regarding interpretability, we agree that a regression forest is less transparent than a simple calendar rule and that individual predictions cannot be explained as directly as those generated by NR. We report variable-importance measures and calibration, and provide the model specification, hyperparameter settings, and analytical code to facilitate reproducibility and independent evaluation. We also emphasize that the apparent precision of an individualized predicted date should not be interpreted as certainty regarding the actual timing of delivery.

Regarding overfitting, we have made the safeguards used during model development and evaluation more explicit. The data were divided into independent training, validation, and test samples; hyperparameters were selected exclusively using the validation sample; and final predictive performance was assessed only in the held-out test sample of 120,771 pregnancies. The same fitted model was additionally evaluated across birth years and geographical regions without retraining or recalibration, providing further information about the stability of its performance. Calibration was close to the identity line across the well-populated central prediction range, while larger deviations were observed in the sparsely populated extreme bins (Supplementary Figure S3). We now report this explicitly and emphasize that calibration should be reassessed in external populations before applied use.

We have also expanded our discussion of differential performance and fairness. Improvements over NR were not uniform across maternal subgroups. We additionally evaluated whether fitting separate models within subgroups defined by parity, maternal age, BMI, and nicotine use improved prediction relative to applying the primary ML model to the same pregnancies. Paired bootstrap comparisons showed that subgroup-specific model fitting generally produced no additional improvements, although some differences were statistically significant (Supplementary Tables S6 and S6b). We now highlight both the heterogeneity in predictive performance and the limited additional benefit of subgroup-specific model fitting in the main text, and emphasize that external validation is required,

particularly because the Swedish study population may not represent populations in settings where an alternative to US-based dating could be most useful.

Finally, we have clarified the intended use of the model. We describe it as a potential decision-support tool for planning and counselling in settings where US dating is unavailable, rather than as a replacement for clinical judgement or ultrasound. We state that predictions should not be used mechanically to trigger obstetric interventions and that clinical implementation would require external validation, assessment of calibration and subgroup performance in the target population, and prospective evaluation of potential effects on perinatal outcomes. Responsibility for clinical decisions must remain with healthcare professionals rather than being transferred to a prediction algorithm.

Comment 3:

I would like to see the model used as supplementary material with the paper. The paper states that the presented model may be of use in low-resource settings, so providing this model cost free to the intended users would be prudent. This would also allay some of the questions arising from the conflicting interest statement.

Response 3:

We appreciate this comment and have considered it carefully. We agree with the reviewer's underlying principle that, if the model ultimately proves suitable for use in settings where US dating is unavailable, access should not be restricted by cost, and we also recognize the connection the reviewer raises with our competing interests. We have therefore revised the manuscript to clarify both the availability of the model and our competing-interest statement.

Regarding model availability, we do not believe that distributing the fitted model as a ready-to-use clinical tool alongside the present paper would be appropriate. The model has so far been developed and internally validated only in Swedish register data. It has not undergone external or prospective validation. As now emphasized in the Discussion, the model should therefore not currently be used to guide clinical interventions, and clinical implementation would require external validation, assessment of calibration and subgroup performance in the target population, and prospective evaluation of potential effects on perinatal outcomes. We nevertheless agree that the model should be available for independent scientific evaluation. We provide the complete model specification, including predictor definitions and coding, outcome definition, sample construction, hyperparameter grid and selected configuration, random seed, and software version. In addition, all data-processing and analysis code will be published as supplementary information with the article.

The fitted grf object itself cannot be publicly deposited because it retains individual-level information from the Swedish Medical Birth Register that we are legally and ethically prohibited from sharing. We have therefore constructed a shareable representation of the fitted forest containing the split variables, split thresholds, and terminal-node predictions, without the underlying individual-level register data. Predictions generated from this representation reproduce those of the fitted model to numerical precision. This representation will be made available to researchers on reasonable request, at no cost, under an agreement restricting its use to external validation or methodological evaluation.

We have clarified this arrangement in the Data Availability statement. We have deliberately distinguished access for independent validation, which we wish to facilitate, from distribution as a ready-to-use clinical tool, which we believe would be premature before external validation. Broader clinical implementation will therefore await appropriate external validation. We have also stated our commitment that, should the model ultimately prove suitable for implementation in settings where US dating is unavailable and the requirements for applying the model are met, cost to users should not constitute a barrier to access.

Regarding competing interests, we take seriously the reviewer's concern that the decision not to distribute the model publicly could be interpreted in light of the authors' financial interests. We have therefore expanded the competing-interest statement to disclose that two authors are shareholders in a company marketing due-date predictions, that they previously developed a different prediction model for that company, and that they hold intellectual-property rights to the model reported in this study, which could have future commercial value. We additionally disclose that the third author has no financial interest in the company, had full access to the study data, independently executed the analysis code, and verified the reported results.

The model reported in this study is not currently deployed, licensed, or sold. To increase transparency despite the restrictions on the underlying data and fitted object, we make the analytical code and model specification public and make the shareable representation of the fitted model available at no cost for independent external validation. We believe this approach provides the transparency necessary for independent scrutiny while avoiding distribution of an internally validated model as though it were ready for clinical use.

Comment 4:

The paper is well written and in good English, I have however spotted two typos: Page 5 line 10 duplicate; Page 7 line 28 incomplete sentence.

Response 4:

Thank you. We have corrected the typos.

<u>Reviewer 3</u>

Comment 1:

The population is highly selected, enrolling delivery between 37 and 42 weeks. This may have overlooked the beneficial effect of US as the date of delivery is influenced, if not determined, by the findings of US (especially at later stages of pregnancy), making the accuracy of NR and US similar and the minor improvement of ML models compared to NR.

Response 1:

We thank the reviewer for this important observation. We agree that requiring both NR- and US-based gestational age at delivery to fall between 37 and 42 weeks could reduce the observed difference between the two dating methods. To examine this directly, we conducted an additional sensitivity analysis in which we expanded the held-out evaluation sample to include a random 20% sample of otherwise eligible spontaneous singleton pregnancies that had been excluded solely because NR- or ultrasound-based gestational age fell outside the 37–42-week range. The originally estimated ML model was applied to these additional observations without retraining or retuning.

The sensitivity analysis supports the reviewer's concern regarding the relative performance of NR and US. The difference between ultrasound and the other dating approaches became larger when pregnancies outside the original gestational-age restriction were included.

Importantly, however, the central finding that the ML model improves prediction relative to NR persisted in the expanded evaluation sample.

We have added this analysis as a sensitivity analysis in the Supplementary Material (Table S5) and revised the manuscript to acknowledge explicitly that the primary 37–42-week restriction attenuates the observed advantage of US. Because the ML model was developed within the original restricted population, we emphasize that the model is not trained on preterm and post-term deliveries, where dating accuracy may be more clinically consequential.

Comment 2:

Another important issue is that the small discrepancies of accuracies are neither compared statistically nor discussed clinically.

Response 2:

We agree on both points and have revised the manuscript to address the statistical and clinical interpretation of the observed differences.

Statistical comparison. Because all methods are evaluated in the same independent test set of 120,771 pregnancies, we added paired bootstrap comparisons with 1,000 resamples, thereby accounting for the within-pregnancy dependence between predictions from the different methods. We designate accuracy within ±7 days as the primary performance metric and ML versus NR as the primary comparison, reflecting the intended use of the model in settings where NR rather than US would otherwise be used. Given the large test sample, we emphasize effect estimates and their confidence intervals rather than statistical significance alone. Full paired comparisons are reported in Supplementary Table S4.

Comparison with ultrasound. We additionally report paired comparisons between ML and ultrasound (Supplementary Table S4). In the full sample, ultrasound performs better than ML across all five metrics. We have also extended the paired comparisons to the second-birth analysis, comparing the model incorporating gestational duration from the previous pregnancy directly with NR, US, and the primary ML model on the same evaluation sample (Table 2 and Supplementary Table S4a). The second-birth model performed better than both NR and the primary ML model across all five metrics. The model produced prediction accuracy comparable to, and on some measures slightly better than, US.

Clinical interpretation. We agree that statistical significance alone does not establish clinical importance. We therefore now express the absolute improvements in more interpretable

terms. Compared with NR, the improvement corresponds to approximately one additional correct prediction within ±7 days per 54 pregnancies, one additional correct prediction within ±1 day per 189 pregnancies, and one additional correct exact-day prediction per 527 pregnancies. No established minimum clinically important difference exists for due-date prediction, and we do not attempt to define one. We therefore describe the improvement in the full sample as statistically robust but modest and explicitly acknowledge that its practical importance will depend on the setting and intended use. We have added this interpretation to both the Results and Discussion. We also report that the magnitude of improvement over NR varies across maternal subgroups and, using paired comparisons, show that fitting separate subgroup-specific models generally provides no additional gains over the primary ML model (Supplementary Tables S6 and S6b). In contrast, incorporating gestational duration from the first pregnancy in the second-pregnancy analysis produced a larger improvement over the primary ML model, illustrating that predictive gains depend not only on the population considered but also on the information available for prediction.

Comment 3:

The conclusion may change entirely if ML does not provide significant improvement in accuracy of delivery date, statistically or clinically, compared to NR.

Response 3:

We agree that the conclusion should depend not only on whether the model performs better than NR, but also on the magnitude and potential practical importance of that improvement. As detailed in our response to Comment 2, paired bootstrap comparisons show that ML improves on NR across all five performance metrics in the independent test set.

We agree, however, that this represents a modest average improvement, despite the statistical precision of the estimate. We have therefore revised the manuscript throughout to avoid equating statistical significance with clinical importance. There is no established minimum clinically important difference for due-date prediction, and we do not propose one. Instead, we report the absolute differences and their confidence intervals, translate the principal differences into interpretable frequencies, as well as Gap Closure (comparing the ML accuracy improvement with US as the gold standard), and leave the practical relevance to be considered in relation to the intended setting and use.

The subgroup analyses also show that the magnitude of improvement is heterogeneous. For example, among women with BMI above 30, accuracy within ±7 days increases from 60.6% with NR to 64.5% with ML, corresponding to approximately one additional correct prediction within ±7 days per 26 pregnancies, whereas ML does not consistently improve on NR among women with BMI at or below 18.5. In the separate analysis of second pregnancies incorporating gestational duration from the first pregnancy, accuracy within ±7 days was 70.5%, compared with 65.3% for NR and 67.3% for the primary ML model applied to the same pregnancies. Relative to NR, this corresponds to approximately one additional 19 pregnancies.

Importantly, paired comparisons also showed that incorporating first-pregnancy gestational duration increased

±7-day accuracy by 3.14 percentage points relative to the primary ML model (95% CI 2.79 to 3.45;

p<0.001). We report these results as evidence that the magnitude of improvement varies across populations and according to the information available, rather than as evidence that the model is clinically beneficial in these groups.

We have consequently revised the conclusion to state that incorporating maternal characteristics produces a statistically robust but modest improvement over NR in the primary study population, while US remains more accurate overall. We further emphasize that whether improvements of this magnitude are clinically meaningful cannot be determined from predictive accuracy alone and would require evaluation of consequences for clinical decision-making and perinatal outcomes. External validation is also required before applied use.

Reviewer 4

Comment 1:

Ultrasound predictions inherently rely on diverse statistical and machine-learning models to calculate biometric estimates. Because ultrasound accuracy has evolved over time, the study would be more significant if it analyzed how historical improvements in US technology compare to this new ML model. Without this baseline, the exact scientific contribution of the study is difficult to measure.

Response 1:

We agree that ultrasound dating has evolved historically through improvements in equipment, biometric reference standards, and clinical implementation. However, the objective of the present study is not to quantify the historical technological development of ultrasound dating. Rather, US serves as the clinical benchmark against which NR and the ML model are compared.

We selected births from 2008–2017 to provide a relatively consistent clinical benchmark for comparing the dating methods. To address the reviewer's concern empirically, we have now examined the predictive performance of US, NR, and ML across five consecutive two-year birth-period groups (2008–2009, 2010–2011, 2012–2013, 2014–2015, and 2016–2017). The results show that US remained the most accurate of the three methods throughout the study period across exact-day, ±1-day, and ±7-day accuracy. Although performance varied slightly across periods, there was no indication of a systematic improvement in US prediction accuracy over 2008–2017 that would materially alter its role as the clinical benchmark in our comparison. These results are reported in Supplementary Figure S5 and described in Section 3.3.

We have also clarified the rationale for the study period in the Methods. We agree that studying the historical evolution of US dating could be informative, but such an analysis would address a different research question and would require data spanning meaningful changes in ultrasound equipment, biometric standards, and clinical practice. Our contribution is instead to ask whether routinely available maternal characteristics can improve upon NR when evaluated against US dating during a period of established routine use.

Comment 2:

The study relies entirely on a single dataset from a specific geographic and demographic population (Sweden). A universal conclusion cannot be drawn from a single data source, as it lacks external validation. Furthermore, the ML model's accuracy may have been artificially

limited by the software used; utilizing a general-purpose prediction software may have yielded lower/higher accuracy than a specialized, medically optimized algorithm.

Response 2:

We agree with both points and have revised the manuscript accordingly.

Single data source and external validation. Following TRIPOD+AI, we now describe the study explicitly as the development and internal validation of a prediction model and have removed wording that could imply universal applicability. The model was developed and evaluated entirely using data from the Swedish Medical Birth Register and has not been externally validated. We now state explicitly in the Abstract and Discussion that performance may differ in different, and that external validation and potentially recalibration are required before use in other populations. We have also added analyses of performance across birth periods and geographical regions within the independent test set (Supplementary Figures S5 and S6). These analyses provide evidence on the stability of performance within the Swedish data but, as we emphasize in the manuscript, do not constitute external validation or establish transportability.

Choice of prediction method. We have also clarified the rationale for using a regression forest. We selected this approach because it accommodates nonlinear relationships and interactions among maternal characteristics without imposing a prespecified functional form, while retaining useful features for interpretation and reproducibility, including variable-importance measures and a reproducible tree-based model structure. The model was implemented using the grf package, which implements the generalized random forest framework of Athey, Tibshirani and Wager (2019), and hyperparameters were selected using a separate validation sample. Predictive performance is determined by the fitted estimator and its specification rather than by whether the software is general-purpose or medically specific; clinical domain knowledge enters through choices such as the predictors, outcome, study population, and prediction target.

We agree, however, with the reviewer's underlying point that the choice of prediction method can affect predictive performance. We did not systematically compare the regression forest with alternative model classes, such as penalised regression, gradient boosting, or neural networks, and therefore do not claim that the selected estimator provides the maximum predictive accuracy attainable from these predictors. We now state this explicitly as a limitation. Our conclusions are consequently restricted to the performance of the model developed and evaluated here, rather than to machine-learning methods generally.

We have also added to the Discussion that future work could systematically compare alternative model classes and prediction targets. The present model minimises squared prediction error and produces a point prediction of delivery timing. Alternative formulations—for example, prediction intervals or models targeting the probability of delivery within clinically relevant windows—may ultimately be more useful for clinical applications.

Comment 3:

Because ultrasound dating is not 100% perfect, a more valuable research objective for the machine learning model would have been investigating how incorporating maternal characteristics directly into ultrasound data could improve overall US accuracy. Enhancing ultrasound precision represents a more meaningful clinical advancement than simply optimizing the less reliable Naegele's Rule.

Response 3:

We agree that combining maternal characteristics with fetal biometric measurements is an important and potentially valuable direction for improving US-based dating. This would, however, address a different use case from the one motivating the present study. Our primary research question is whether routinely available maternal characteristics can improve due-date prediction relative to NR in settings where US information is unavailable. A prediction model requiring fetal biometric measurements would not address that setting.

We nevertheless agree with the reviewer that the two approaches are potentially complementary. We have therefore written in the Discussion that future research combining maternal characteristics with US or fetal biometric measurements is a promising direction in settings where US is available.

Such an analysis would ideally use the underlying fetal biometric measurements, scan timing, and information on the dating algorithm or reference standard applied, rather than only the resulting registered US-based EDD. These data are not available for the present analysis.

Comment 4:

The authors argue that this model is highly useful in environments where ultrasound technology is unavailable. However, the proposed regression forest model is a complex, computer-intensive algorithm that cannot be performed manually. In short, the model requires advanced digital infrastructure to run, yet it yields less accurate results than an ultrasound. This creates a practical paradox, making it unusable in the very low-resource areas it claims to benefit.

Response 4:

We appreciate this comment, which identifies an ambiguity in our original presentation that we have now corrected. The apparent paradox arises from an important distinction between the resources required to develop the model and those required to apply an already fitted model.

Model development required a large register dataset, substantial computing capacity, and a validation infrastructure. These requirements would not need to be replicated when applying a fixed, validated model. Once fitted, a regression forest produces a prediction through a sequence of threshold comparisons on the seven maternal characteristics followed by aggregation across trees.

The computational requirements for generating an individual prediction are therefore modest and compatible with implementation on ordinary digital devices, such as a laptop or mobile phone with or without internet connection.

We agree, however, that computational feasibility does not by itself make the model suitable for use in low-resource settings. Implementation would additionally require access to the necessary maternal information and basic digital infrastructure, as well as external validation and, where necessary, recalibration in the intended population. We have therefore revised the manuscript to qualify references to low-resource settings accordingly.

The resource requirements also differ fundamentally from those of obstetric ultrasound, which requires dedicated equipment and appropriately trained personnel. We nevertheless agree that ultrasound remains more accurate overall in our data and should remain the preferred dating method where it is available. The relevant comparison for the intended use case of the present model is therefore with NR in settings where US is unavailable.

We have added a short paragraph to the Discussion distinguishing the resources required for model development from those required for application and have revised the Abstract and other statements about the intended setting to avoid implying that computational simplicity alone establishes feasibility or clinical utility. As emphasized elsewhere in the revised manuscript, the model has not been externally validated and is not currently distributed as a ready-to-use clinical tool.

VERSION 2 - REVIEW

**Reviewer**	**2**
**Name**	**Sande, Ragnar**
**Affiliation**	**Stavanger University Hospital, Department of Obstetrics and Gynaecology**
**Date**	**23-Sep-2026**
**COI**	**None**

The pape is well written and the limitations are thorougly discussed. My only issue with the paper as it is would be a tendency to overuse abbreviations. I would like to see a lot less abbreviations, but at least ML (Machine Learning), US (Ultrasound) and NR (Naegeles rule) need to be fully written out for. I would further recommend abbreviations to be resticted to generally established terms that occur at least five times in the text.

VERSION 2 - AUTHOR RESPONSE

Reviewer: 2

Dr. Ragnar Sande, Stavanger University Hospital

Comments to the Author: The paper is well written and the limitations are thorougly discussed. My only issue with the paper as it is would be a tendency to overuse abbreviations. I would like to see a lot less abbreviations, but at least ML (Machine Learning), US (Ultrasound) and NR (Naegeles rule) need to be fully written out for. I would further recommend abbreviations to be resticted to generally established terms that occur at least five times in the text.

Response: Thank you for this helpful suggestion. We agree that the extensive use of abbreviations could reduce readability. We have therefore reviewed the manuscript throughout and substantially reduced the use of abbreviations. In particular, we have removed the abbreviations ML, US and NR and now write out machine learning, ultrasound and Naegele's rule, respectively. We have also removed other abbreviations that were not generally established or were used infrequently, retaining abbreviations only where they are well established and used repeatedly in the manuscript
