# Supplementary material for: Can maternal characteristics improve due date prediction without ultrasound? Development and internal validation of a machine-learning prediction model in 600 000 pregnancies in Sweden
Source: BMJ Open. 2026 Oct 23;16(10):e120970. doi: 10.1136/bmjopen-2026-120970 (PMC13711609; doi:10.1136/bmjopen-2026-120970)
Supplement: Supplementary file 2 [file bmjopen-16-10-s001.docx]

**Supplementary Material for “Can maternal characteristics improve due date prediction without ultrasound? Development and internal validation of a machine-learning prediction model in 600,000 pregnancies in Sweden”**

**Gilson Dutra**^1,+^**, Mikael Elinder**^1,+^**, and Oscar Erixson (Corresponding author)**^2,*,+^

^1^Department of Economics, Uppsala University, Uppsala, 751 05, Sweden

^2^Institute for Housing and Urban Research, Uppsala University, Uppsala, 751 05, Sweden ^*^oscar.erixson@ibf.uu.se

^+^these authors contributed equally to this work

**Appendix**

# A. Inclusion criteria

Table S1 summarizes the number of observations remaining after each inclusion step. The initial dataset contained 1,120,704 pregnancies recorded in the Swedish Medical Birth Register between 2008 and 2017. Before applying the main inclusion criteria, we excluded pregnancies with incomplete or missing information on the last menstrual period, multiple births, births occurring outside hospital settings, and repeated gestations within the same calendar year for the same mother (a very small number of cases). After these preliminary exclusions, 1,005,775 singleton births remained.

We then applied a set of restrictions to ensure comparability across dating methods and to focus the primary analysis on spontaneous term pregnancies:

- Exclusion of stillbirths (3,397 cases);
- Exclusion of neonatal deaths within 0–6 days (796 cases);
- Exclusion of neonatal deaths within 7–27 days (316 cases);
- Exclusion of non-spontaneous deliveries, including medically induced deliveries and planned cesarean sections (233,285 cases);
- Requirement of a completed routine second-trimester ultrasound examination (32,933 cases);
- Exclusion of pregnancies with missing data on key maternal predictors (22,925 cases);
- Restriction to pregnancies with both Naegele’s rule- and ultrasound-based gestational duration between 37 and 42 completed weeks (108,262 cases).

**Table S1.** Number of observations remaining after each inclusion step

| Criterion | Remaining observations | Excluded cases |
| --- | --- | --- |
| Initial singleton pregnancies (2008–2017) | 1,005,775 | – |
| Stillbirths excluded | 1,002,378 | 3,397 |
| Neonatal deaths 0–27 days excluded | 1,001,266 | 1,112 |
| Non-spontaneous deliveries excluded | 767,981 | 233,285 |
| Without routine ultrasound excluded | 735,048 | 32,933 |
| Missing predictor data excluded | 712,123 | 22,925 |
| Gestational duration outside 37–42 weeks excluded | 603,861 | 108,262 |
| Final analytical sample | 603,861 |  |

Data source: Swedish Medical Birth Register (*Medicinska födelseregistret*), 2008–2017.

For the final restriction, both Naegele’s rule- and ultrasound-based gestational age at delivery were required to fall between 37 and 42 completed weeks for the same pregnancy. This restriction follows the focus on term spontaneous deliveries used in previous studies of expected due date accuracy but may affect the observed relative performance of Naegele’s rule and ultrasound by excluding pregnancies in which the two dating methods differ sufficiently for one or both estimates to fall outside this range. We therefore examine the sensitivity of the results to this restriction in an expanded evaluation sample (Section D, Table S5).

The final analytic sample consists of 603,861 term singleton pregnancies with complete maternal and gestational information, allowing for rigorous comparison of Naegele’s rule-, ultrasound-, and machine-learning-based estimated due dates to be compared under identical inclusion criteria.

# B. Summary statistics for training, validation, and test data

Table S2 reports summary statistics for the training, validation, and independent test subsets used for model development and internal validation. The distributions of all predictors are nearly identical across the three randomly partitioned subsets. A fixed random seed (846) was used to ensure reproducibility of the train–validation–test partition. The validation subset was used for hyperparameter selection, whereas the test subset was reserved for final evaluation and was not used for model fitting or hyperparameter selection.

**Table S2.** Summary statistics for training, validation, and test data

Training (*N* = 362*,*319) Validation (*N* = 120*,*771) Test (*N* = 120*,*771)

| Variable | Mean | St. Dev. | Mean | St. Dev. | Mean | St. Dev. |
| --- | --- | --- | --- | --- | --- | --- |
| Naegele’s rule-based GA (days) | 280.900 | 7.628 | 280.889 | 7.637 | 280.900 | 7.629 |
| Ultrasound-based GA (days) | 280.142 | 7.354 | 280.127 | 7.367 | 280.151 | 7.339 |
| Parity | 0.816 | 0.896 | 0.817 | 0.898 | 0.822 | 0.900 |
| Maternal age (years) | 29.756 | 5.016 | 29.763 | 5.013 | 29.773 | 5.009 |
| Maternal weight (kg) | 67.377 | 12.837 | 67.327 | 12.815 | 67.342 | 12.828 |
| Maternal height (cm) | 166.298 | 6.415 | 166.273 | 6.433 | 166.289 | 6.441 |
| Nicotine use (share) | 0.168 | 0.374 | 0.169 | 0.375 | 0.166 | 0.372 |
| Hypertension (share) | 0.003 | 0.057 | 0.003 | 0.057 | 0.003 | 0.056 |
| Diabetes (share) | 0.003 | 0.055 | 0.003 | 0.056 | 0.003 | 0.056 |

Note: “GA” stands for gestational age. Data source: Swedish Medical Birth Register (Medicinska födelseregistret), 2008−2017.

# C. Model specifications

All machine-learning models were estimated using the grf package (version 2.4.0) in R (version 4.4.2). Hyperparameters were selected via grid search using the validation dataset, with mean squared error (MSE) as the tuning criterion.

**Hyperparameter grid.**	The following values were considered:

- num.trees: 100, 1000
- sample.fraction: 0.10, 0.20, 0.30, 0.40, 0.50
- mtry: 2, 3, 4
- alpha: 0.05, 0.10, 0.15, 0.20
- min.node.size: 100, 500, 1000

This yielded a total of 360 candidate models.

**Selected configuration.**	The model with the lowest validation-set MSE used the following hyperparameters:

- num.trees = 100
- sample.fraction = 0.50
- mtry = 4
- alpha = 0.05
- min.node.size = 100

All remaining parameters were kept at their grf defaults; honesty.fraction, honesty.prune.leaves, and imbalance.penalty were not modified. Hyperparameters were selected exclusively using the validation subset, and final predictive performance was evaluated only in the held-out test subset.

We selected a regression forest because it accommodates nonlinear relationships and interactions among predictors without requiring prespecified functional forms, while retaining a reproducible tree-based structure and providing measures of variable importance. We did not systematically compare alternative model classes and therefore do not claim that the regression forest is the optimal estimator for this prediction problem.

# D. Additional Results

**Figure S1.** Regression Forest Tree

Note: Example of a simplified decision tree, where end nodes (leaves) show the conditional expectation of gestational duration (in bold). Regression forests partition continuous variables using threshold-based splits, and the apparent categorization in schematic trees does not imply loss of continuous information.

**Figure S2.** Predictor importance

Note: ML refers to machine-learning. Variable importance scores were computed using the default honesty-based splitting frequencies implemented in the grf package, reflecting how often each variable was selected for splits across the ensemble.

**Figure S3.** Machine-learning prediction vs observed last menstrual period

Note: Model calibration by predicted gestational duration (days). Points show mean observed gestational age at delivery within each day-level prediction bin; the dashed 45-degree line indicates perfect calibration. Calibration is close to the identity line across the well-populated central prediction range, with larger deviations in sparsely populated extreme bins.

**Figure S4.** Variable Importance for Model Based on Primiparous Women

Note: GL refers to gestational length. Variable importance scores were computed using the default honesty-based splitting frequencies implemented in the grf package, reflecting how often each variable was selected for splits across the ensemble.

**Figure S5.** Prediction accuracy of machine-learning model, Naegele’s rule, and ultrasound across two-year birth periods

Note: Predictive accuracy in the independent test set, stratified by two-year birth periods (2008–2009, 2010–2011, 2012–2013, 2014–2015, and 2016–2017). Points show exact-day accuracy, accuracy within ±1 day, and accuracy within ±7 days for the machine-learning model (ML), Naegele’s rule (NR), and ultrasound-based dating (US). Error bars indicate 95% confidence intervals obtained by non-parametric bootstrap within each birth-period stratum. The same fitted ML model was applied in all periods without retraining or recalibration.

**Figure S6.** Prediction accuracy of machine-learning model, Naegele’s rule, and ultrasound across geographical regions

Note: Predictive accuracy in the independent test set, stratified by geographical region (Götaland, Svealand, and Norrland). Points show exact-day accuracy, accuracy within ±1 day, and accuracy within ±7 days for the machine-learning model (ML), Naegele’s rule (NR), and ultrasound-based dating (US). Error bars indicate 95% confidence intervals obtained by non-parametric bootstrap within each regional stratum. The same fitted ML model was applied in all regions without retraining or recalibration.

**Table S3.** Distribution of actual and predicted delivery timing relative to last menstrual period (LMP) in the independent test set (N = 120,771)

| **Quantity** | **Mean (95% CI)** | **Median** | **IQR** |
| --- | --- | --- | --- |
| Actual delivery (days from LMP) | 280.900 (280.857-280.943) | 282.000 | 276-287 |
| Naegele’s rule prediction (days from LMP) | 280.000 (fixed) | 280.000 | 280-280 |
| Ultrasound prediction (days from LMP) | 280.751 (280.722-280.781) | 280.000 | 278-284 |
| Machine-learning prediction (days from LMP) | 280.901 (280.897-280.905) | 280.955 | 280.510-281.349 |

Note: All quantities are expressed as days from the reported first day of the last menstrual period (LMP). Actual delivery denotes the observed date of delivery expressed on this scale. Naegele’s rule prediction is fixed at 280 days from LMP by definition. Ultrasound prediction denotes the ultrasound-predicted due date expressed relative to LMP, and machine-learning prediction denotes the delivery timing predicted by the machine-learning model. Values in parentheses are 95% CIs for the mean. IQR denotes interquartile range. Data source: Swedish Medical Birth Register (*Medicinska födelseregistret*), 2008–2017.

**Table S4.** Paired differences in predictive performance between machine-learning (ML), Naegele’s rule (NR), and ultrasound (US) in the independent test set (N = 120,771)

| **Model comparisons** | **Metric** | **Accuracy difference** | **95% CI** | **p-value** |
| --- | --- | --- | --- | --- |
| ML vs. NR | Exact day | +0.190 pp | +0.027 to +0.350 | 0.022 |
|  | Within ±1 day | +0.528 pp | +0.359 to +0.710 | <0.001 |
|  | Within ±7 days | +1.865 pp | +1.719 to +2.005 | <0.001 |
|  | MSE | -1.204 days^2^ | -1.299 to -1.106 | <0.001 |
|  | RMSE | -0.079 days | -0.085 to -0.072 | <0.001 |
| ML vs. US | Exact day | -0.415 pp | -0.590 to -0.255 | <0.001 |
|  | Within ±1 day | -0.953 pp | -1.206 to -0.672 | <0.001 |
|  | Within ±7 days | -2.199 pp | -2.487 to -1.908 | <0.001 |
|  | MSE | +3.072 days^2^ | +1.254 to +4.189 | 0.018 |
|  | RMSE | +0.205 days | +0.083 to +0.280 | 0.018 |

Note: Differences were calculated within the same independent test set and estimated using paired non-parametric bootstrap resampling with 1,000 replications. For exact-day, ±1-day, and ±7-day accuracy, positive differences indicate higher accuracy for the first method; for MSE and RMSE, negative differences indicate lower prediction error for the first method. CI, confidence interval; pp, percentage points; ML, machine learning; NR, Naegele’s rule; US, ultrasound-based dating; MSE, mean squared error; RMSE, root mean squared error. Data source: Swedish Medical Birth Register (*Medicinska födelseregistret*), 2008–2017.

**Table S4a.** Paired differences in predictive performance between machine-learning (ML), Naegele’s rule (NR), and ultrasound (US), among second pregnancies in the independent test set (N = 40,781)

| **Model** **comparisons** | **Metric** | **Difference** | **95% CI** | **p-value** |
| --- | --- | --- | --- | --- |
| ML vs. NR | Exact day | +0.078 pp | -0.186 to +0.378 | 0.585 |
|  | Within ±1 day | +0.564 pp | +0.262 to +0.873 | 0.003 |
|  | Within ±7 days | +2.089 pp | +1.839 to +2.364 | <0.001 |
|  | MSE | -2.027 days^2^ | -2.177 to -1.855 | <0.001 |
|  | RMSE | -0.136 days | -0.147 to -0.125 | <0.001 |
| ML vs. US | Exact day | -0.441 pp | -0.728 to -0.150 | 0.003 |
|  | Within ±1 day | -0.802 pp | -1.253 to -0.343 | 0.002 |
|  | Within ±7 days | -2.298 pp | -2.764 to -1.805 | <0.001 |
|  | MSE | +3.573 days^2^ | +3.013 to +4.207 | <0.001 |
|  | RMSE | +0.247 days | +0.209 to +0.290 | <0.001 |
| ML vs. ML(SB) | Exact day | -0.490 pp | -0.780 to -0.198 | <0.001 |
|  | Within ±1 day | -1.290 pp | -1.670 to -0.878 | <0.001 |
|  | Within ±7 days | -3.136 pp | -3.445 to -2.793 | <0.001 |
|  | MSE | +4.389 days^2^ | +4.088 to +4.689 | <0.001 |
|  | RMSE | +0.304 days | +0.284 to +0.325 | <0.001 |
| ML(SB) vs. NR | Exact day | +0.569 pp | +0.250 to +0.868 | <0.001 |
|  | Within ±1 day | +1.854 pp | +1.417 to +2.278 | <0.001 |
|  | Within ±7 days | +5.225 pp | +4.855 to +5.574 | <0.001 |
|  | MSE | -6.415 days^2^ | -6.753 to -6.096 | <0.001 |
|  | RMSE | -0.441 days | -0.463 to -0.419 | <0.001 |
| ML(SB) vs. US | Exact day | +0.049 pp | -0.265 to +0.361 | 0.754 |
|  | Within ±1 day | +0.488 pp | -0.015 to +0.939 | 0.049 |
|  | Within ±7 days | +0.839 pp | +0.341 to +1.312 | <0.001 |
|  | MSE | -0.816 days^2^ | -1.405 to -0.174 | 0.016 |
|  | RMSE | -0.058 days | -0.099 to -0.012 | 0.016 |

Note: Differences were calculated within the same independent test set of second births and estimated using paired non-parametric bootstrap resampling with 1,000 replications. For exact-day, ±1-day, and ±7-day accuracy, positive differences indicate higher accuracy for the first method; for MSE and RMSE, negative differences indicate lower prediction error for the first method. CI, confidence interval; pp, percentage points; ML, machine-learning model; ML(SB), second-birth machine-learning model incorporating gestational duration from the previous pregnancy; NR, Naegele’s rule; US, ultrasound-based dating; MSE, mean squared error; RMSE, root mean squared error. Data source: Swedish Medical Birth Register (*Medicinska födelseregistret*), 2008–2017.

**Table S5.** Sensitivity analysis of prediction performance of machine-learning model (ML), Naegele’s rule (NR), and ultrasound (US) in an expanded evaluation sample including pregnancies outside the 37–42-week restriction

|  | Exact Day | ±1 Day | ±7 Days | MSE | RMSE |
| --- | --- | --- | --- | --- | --- |
| ML | 4.27% [4.17, 4.37] | 12.69% [12.52, 12.86] | 56.94% [56.66, 57.21] | 144.42 [141.83, 147.22] | 12.02 [11.91, 12.13] |
| NR | 4.11% [4.01, 4.21] | 12.23% [12.07, 12.39] | 55.28% [55.01, 55.53] | 145.98 [143.43, 148.72] | 12.08 [11.98, 12.20] |
| US | 4.82% [4.70, 4.93] | 14.07% [13.88, 14.26] | 61.82% [61.56, 62.06] | 115.12 [112.33, 118.02] | 10.73 [10.60, 10.86] |
| Gap Closure | 22.98% | 25.14% | 25.27% | 5.04% | 4.77% |

Note: The expanded evaluation sample consists of the original independent test set supplemented with a random 20% sample of otherwise eligible spontaneous singleton pregnancies that were excluded from the primary analytical sample solely because NR-based gestational age, US-based gestational age, or both fell outside the 37–42-week range. The previously trained ML model was applied to these additional observations without retraining or hyperparameter retuning. ML, machine learning; NR, Naegele’s rule; US, ultrasound-based dating; MSE, mean squared error; RMSE, root mean squared error. Data source: Swedish Medical Birth Register (*Medicinska födelseregistret*), 2008–2017.

**Table S6.** Performance of tailored machine learning models across subgroups

| Parity = 0 | Exact Day | ±1 Day | ±7 Days | MSE | RMSE |
| --- | --- | --- | --- | --- | --- |
| ML(SG) Accuracy | 4.79% [4.59, 4.98] | 14.33% [14.02, 14.63] | 64.75% [64.34, 65.16] | 58.96 [58.43, 59.71] | 7.68 [7.64, 7.73] |
| NR Accuracy | 4.56% [4.40, 4.74] | 13.63% [13.34, 13.94] | 62.35% [61.94, 62.76] | 61.08 [60.46, 61.72] | 7.82 [7.78, 7.86] |
| US Accuracy | 5.00% [4.81, 5.19] | 14.72% [14.42, 15.03] | 65.83% [65.43, 66.24] | 57.31 [56.67, 57.91] | 7.57 [7.53, 7.61] |
| Gap Closure | 51.33% | 64.31% | 68.97% | 56.07% | 55.68% |
| Parity ≥ 1 | Exact Day | ±1 Day | ±7 Days | MSE | RMSE |
| ML(SG) Accuracy | 5.20% [5.01, 5.40] | 15.46% [15.12, 15.79] | 67.14% [66.71, 67.56] | 54.96 [54.39, 55.73] | 7.41 [7.37, 7.46] |
| NR Accuracy | 4.91% [4.71, 5.10] | 14.68% [14.35, 14.99] | 64.92% [64.47, 65.35] | 57.01 [56.35, 57.68] | 7.55 [7.51, 7.59] |
| US Accuracy | 5.30% [5.11, 5.52] | 16.04% [15.72, 16.37] | 69.20% [68.79, 69.65] | 51.44 [50.83, 52.03] | 7.17 [7.13, 7.21] |
| Gap Closure | 73.54% | 57.81% | 51.86% | 36.88% | 36.29% |
| Age ≤ 30 | Exact Day | ±1 Day | ±7 Days | MSE | RMSE |
| ML(SG) Accuracy | 4.96% [4.80, 5.13] | 14.72% [14.45, 14.97] | 65.76% [65.40, 66.16] | 57.21 [56.69, 57.87] | 7.56 [7.53, 7.61] |
| NR Accuracy | 4.64% [4.48, 4.80] | 14.11% [13.84, 14.38] | 63.36% [63.02, 63.77] | 59.55 [58.97, 60.09] | 7.72 [7.68, 7.75] |
| US Accuracy | 5.31% [5.14, 5.49] | 15.76% [15.48, 16.04] | 68.07% [67.73, 68.44] | 55.69 [53.79, 59.06] | 7.46 [7.33, 7.68] |
| Gap Closure | 48.03% | 36.81% | 51.01% | 60.55% | 60.15% |
| Age > 30 | Exact Day | ±1 Day | ±7 Days | MSE | RMSE |
| ML(SG) Accuracy | 5.06% [4.88, 5.26] | 15.00% [14.70, 15.32] | 66.42% [66.01, 66.84] | 56.65 [56.09, 57.39] | 7.53 [7.49, 7.58] |
| NR Accuracy | 4.75% [4.56, 4.93] | 14.38% [14.07, 14.71] | 64.66% [64.26, 65.08] | 58.39 [57.74, 58.98] | 7.64 [7.60, 7.68] |
| US Accuracy | 5.17% [5.00, 5.36] | 15.29% [15.00, 15.60] | 67.14% [66.74, 67.56] | 54.34 [53.79, 54.95] | 7.37 [7.33, 7.41] |
| Gap Closure | 73.78% | 68.53% | 70.95% | 42.86% | 42.42% |
| BMI ≤ 18.5 | Exact Day | ±1 Day | ±7 Days | MSE | RMSE |
| ML(SG) Accuracy | 5.52% [4.74, 6.30] | 15.14% [13.97, 16.47] | 65.01% [63.38, 66.67] | 57.88 [55.66, 60.54] | 7.61 [7.46, 7.78] |
| NR Accuracy | 5.82% [5.07, 6.67] | 14.66% [13.45, 15.90] | 64.10% [62.44, 65.70] | 59.51 [57.10, 62.08] | 7.71 [7.56, 7.88] |
| US Accuracy | 5.07% [4.31, 5.76] | 15.57% [14.27, 16.74] | 66.67% [64.95, 68.21] | 57.12 [54.68, 59.99] | 7.56 [7.39, 7.75] |
| Gap Closure | 40.00% | 53.33% | 35.29% | 68.26% | 68.04% |
| 18.5 < BMI ≤ 24.5 | Exact Day | ±1 Day | ±7 Days | MSE | RMSE |
| ML(SG) Accuracy | 5.00% [4.84, 5.16] | 14.89% [14.63, 15.14] | 66.30% [65.96, 66.63] | 56.68 [56.30, 57.33] | 7.53 [7.50, 7.57] |
| NR Accuracy | 4.68% [4.54, 4.83] | 14.41% [14.14, 14.67] | 64.58% [64.22, 64.90] | 58.20 [57.71, 58.71] | 7.63 [7.60, 7.66] |
| US Accuracy | 5.31% [5.15, 5.47] | 15.58% [15.32, 15.82] | 68.01% [67.67, 68.32] | 53.28 [52.80, 53.78] | 7.30 [7.27, 7.33] |
| Gap Closure | 51.29% | 41.26% | 50.22% | 30.87% | 30.40% |
| 24.5 < BMI ≤ 30.0 | Exact Day | ±1 Day | ±7 Days | MSE | RMSE |
| ML(SG) Accuracy | 5.04% [4.79, 5.27] | 14.66% [14.24, 15.08] | 65.82% [65.29, 66.33] | 57.06 [56.21, 57.92] | 7.55 [7.50, 7.61] |
| NR Accuracy | 4.53% [4.30, 4.76] | 13.82% [13.44, 14.22] | 63.07% [62.54, 63.58] | 59.61 [58.81, 60.39] | 7.72 [7.67, 7.77] |
| US Accuracy | 5.09% [4.85, 5.33] | 15.30% [14.92, 15.69] | 67.48% [66.95, 67.98] | 54.05 [53.23, 54.87] | 7.35 [7.30, 7.41] |
| Gap Closure | 90.06% | 56.70% | 62.38% | 45.86% | 45.26% |
| BMI > 30.0 | Exact Day | ±1 Day | ±7 Days | MSE | RMSE |
| ML(SG) Accuracy | 4.73% [4.36, 5.13] | 14.17% [13.60, 14.76] | 65.31% [64.45, 66.14] | 59.02 [57.69, 60.55] | 7.68 [7.60, 7.78] |
| NR Accuracy | 4.27% [3.93, 4.61] | 13.08% [12.49, 13.68] | 60.64% [59.74, 61.47] | 63.02 [61.66, 64.38] | 7.94 [7.85, 8.02] |
| US Accuracy | 4.72% [4.36, 5.07] | 14.59% [13.99, 15.19] | 65.67% [64.84, 66.42] | 57.56 [56.34, 58.83] | 7.59 [7.51, 7.67] |
| Gap Closure | 103.51% | 72.25% | 92.95% | 73.19% | 72.74% |
| Nicotine Yes | Exact Day | ±1 Day | ±7 Days | MSE | RMSE |
| ML(SG) Accuracy | 4.86% [4.55, 5.16] | 14.63% [14.13, 15.12] | 65.29% [64.65, 65.91] | 58.41 [57.41, 59.55] | 7.64 [7.58, 7.72] |
| NR Accuracy | 4.66% [4.37, 4.95] | 13.78% [13.31, 14.27] | 62.76% [62.07, 63.42] | 61.00 [59.99, 62.06] | 7.81 [7.75, 7.88] |
| US Accuracy | 5.20% [4.90, 5.53] | 15.30% [14.85, 15.84] | 66.98% [66.30, 67.56] | 56.25 [55.28, 57.33] | 7.50 [7.43, 7.57] |
| Gap Closure | 36.94% | 55.52% | 59.95% | 54.56% | 54.05% |
| Nicotine No | Exact Day | ±1 Day | ±7 Days | MSE | RMSE |
| ML(SG) Accuracy | 5.04% [4.89, 5.18] | 14.89% [14.66, 15.10] | 66.26% [65.97, 66.53] | 56.49 [56.12, 57.05] | 7.52 [7.49, 7.55] |
| NR Accuracy | 4.76% [4.64, 4.90] | 14.28% [14.05, 14.50] | 64.19% [63.91, 64.48] | 58.48 [58.04, 58.90] | 7.65 [7.62, 7.67] |
| US Accuracy | 5.22% [5.08, 5.34] | 15.47% [15.27, 15.70] | 67.84% [67.56, 68.13] | 53.73 [53.28, 54.13] | 7.33 [7.30, 7.36] |
| Gap Closure | 60.66% | 51.46% | 56.55% | 41.86% | 41.35% |

Note: Performance results are reported for subgroups defined by parity, maternal age, BMI, and nicotine use. For each subgroup, a separate ML(SG) model was trained using observations belonging to that subgroup. These analyses therefore assess the potential value of subgroup-specific model development rather than subgroup performance of the primary ML model. Performance estimates were calculated using the corresponding subgroup observations in the independent test set. Sample sizes were: Parity = 0 (N = 51,579); Parity ≥ 1 (N = 47,607); Age ≤ 30 (N = 67,493); Age > 30 (N = 53,278); BMI ≤ 18.5 (N = 3,315); 18.5 < BMI ≤ 24.5 (N = 74,431); 24.5 < BMI ≤ 30.0 (N = 30,317); BMI > 30.0 (N = 12,702); Nicotine Yes (N = 20,252); and Nicotine No (N = 100,517). CI, confidence interval; ML(SG), subgroup-specific machine-learning model; NR, Naegele’s rule; US, ultrasound-based dating; MSE, mean squared error; RMSE, root mean squared error. Data source: Swedish Medical Birth Register (Medicinska födelseregistret), 2008–2017.

**Table S6b.** Paired differences in predictive performance across subgroups in the independent test set

| **Subgroup** **analysis** | **Model** **comparisons** | **Metric** | **Difference** | **95% CI** | **p-value** |
| --- | --- | --- | --- | --- | --- |
| Parity = 0 | ML vs. NR | Exact day | +0.225 pp | -0.031 to +0.461 | 0.069 |
|  |  | Within ±1 day | +0.706 pp | +0.436 to +0.962 | <0.001 |
|  |  | Within ±7 days | +2.404 pp | +2.183 to +2.643 | <0.001 |
|  |  | MSE | -2.113 days^2^ | -2.265 to -1.962 | <0.001 |
|  |  | RMSE | -0.136 days | -0.146 to -0.126 | <0.001 |
| Parity = 0 | ML vs. US | Exact day | -0.213 pp | -0.487 to +0.035 | 0.110 |
|  |  | Within ±1 day | -0.392 pp | -0.795 to +0.000 | 0.056 |
|  |  | Within ±7 days | -1.082 pp | -1.516 to -0.653 | <0.001 |
|  |  | MSE | +1.656 days^2^ | +1.063 to +2.243 | <0.001 |
|  |  | RMSE | +0.109 days | +0.070 to +0.147 | <0.001 |
| Parity = 0 | ML vs. ML(SG) | Exact day | +0.058 pp | -0.062 to +0.178 | 0.363 |
|  |  | Within ±1 day | +0.074 pp | -0.043 to +0.190 | 0.226 |
|  |  | Within ±7 days | +0.304 pp | +0.194 to +0.409 | <0.001 |
|  |  | MSE | -0.840 days^2^ | -0.880 to -0.802 | <0.001 |
|  |  | RMSE | -0.055 days | -0.057 to -0.052 | <0.001 |
| Parity = 0 | ML(SG) vs. NR | Exact day | +0.167 pp | -0.068 to +0.394 | 0.178 |
|  |  | Within ±1 day | +0.632 pp | +0.355 to +0.903 | <0.001 |
|  |  | Within ±7 days | +2.100 pp | +1.879 to +2.336 | <0.001 |
|  |  | MSE | -1.273 days^2^ | -1.429 to -1.112 | <0.001 |
|  |  | RMSE | -0.082 days | -0.092 to -0.072 | <0.001 |
| Parity = 0 | ML(SG) vs. US | Exact day | -0.271 pp | -0.537 to -0.010 | 0.045 |
|  |  | Within ±1 day | -0.465 pp | -0.867 to -0.074 | 0.028 |
|  |  | Within ±7 days | -1.386 pp | -1.824 to -0.954 | <0.001 |
|  |  | MSE | +2.496 days^2^ | +1.901 to +3.085 | <0.001 |
|  |  | RMSE | +0.163 days | +0.124 to +0.202 | <0.001 |
| Parity >= 1 | ML vs. NR | Exact day | +0.292 pp | +0.046 to +0.542 | 0.020 |
|  |  | Within ±1 day | +0.786 pp | +0.504 to +1.065 | <0.001 |
|  |  | Within ±7 days | +2.220 pp | +1.987 to +2.458 | <0.001 |
|  |  | MSE | -2.057 days^2^ | -2.209 to -1.895 | <0.001 |
|  |  | RMSE | -0.137 days | -0.148 to -0.127 | <0.001 |
| Parity >= 1 | ML vs. US | Exact day | -0.105 pp | -0.395 to +0.172 | 0.466 |
|  |  | Within ±1 day | -0.573 pp | -1.019 to -0.157 | 0.008 |
|  |  | Within ±7 days | -2.061 pp | -2.508 to -1.628 | <0.001 |
|  |  | MSE | +3.520 days^2^ | +3.009 to +4.110 | <0.001 |
|  |  | RMSE | +0.241 days | +0.206 to +0.282 | <0.001 |
| Parity >= 1 | ML vs. ML(SG) | Exact day | +0.134 pp | +0.013 to +0.267 | 0.036 |
|  |  | Within ±1 day | +0.170 pp | +0.042 to +0.294 | 0.013 |
|  |  | Within ±7 days | +0.357 pp | +0.252 to +0.456 | <0.001 |
|  |  | MSE | -0.769 days^2^ | -0.803 to -0.735 | <0.001 |
|  |  | RMSE | -0.052 days | -0.054 to -0.049 | <0.001 |
| Parity >= 1 | ML(SG) vs. NR | Exact day | +0.158 pp | -0.090 to +0.401 | 0.213 |
|  |  | Within ±1 day | +0.615 pp | +0.340 to +0.893 | <0.001 |
|  |  | Within ±7 days | +1.863 pp | +1.630 to +2.096 | <0.001 |
|  |  | MSE | -1.288 days^2^ | -1.440 to -1.128 | <0.001 |
|  |  | RMSE | -0.086 days | -0.096 to -0.075 | <0.001 |
| Parity >= 1 | ML(SG) vs. US | Exact day | -0.239 pp | -0.529 to +0.019 | 0.097 |
|  |  | Within ±1 day | -0.744 pp | -1.191 to -0.328 | 0.002 |
|  |  | Within ±7 days | -2.418 pp | -2.865 to -1.974 | <0.001 |
|  |  | MSE | +4.288 days^2^ | +3.751 to +4.890 | <0.001 |
|  |  | RMSE | +0.293 days | +0.257 to +0.333 | <0.001 |
| Age <= 30 | ML vs. NR | Exact day | +0.324 pp | +0.092 to +0.550 | 0.007 |
|  |  | Within ±1 day | +0.606 pp | +0.363 to +0.852 | <0.001 |
|  |  | Within ±7 days | +2.405 pp | +2.176 to +2.599 | <0.001 |
|  |  | MSE | -2.338 days^2^ | -2.470 to -2.189 | <0.001 |
|  |  | RMSE | -0.153 days | -0.162 to -0.143 | <0.001 |
| Age <= 30 | ML vs. US | Exact day | -0.351 pp | -0.593 to -0.130 | 0.004 |
|  |  | Within ±1 day | -1.040 pp | -1.399 to -0.656 | <0.001 |
|  |  | Within ±7 days | -2.310 pp | -2.701 to -1.902 | <0.001 |
|  |  | MSE | +1.523 days^2^ | -1.867 to +3.391 | 0.344 |
|  |  | RMSE | +0.101 days | -0.123 to +0.227 | 0.356 |
| Age <= 30 | ML vs. ML(SG) | Exact day | +0.033 pp | -0.081 to +0.161 | 0.575 |
|  |  | Within ±1 day | +0.098 pp | -0.019 to +0.213 | 0.098 |
|  |  | Within ±7 days | +0.234 pp | +0.132 to +0.329 | <0.001 |
|  |  | MSE | -0.770 days^2^ | -0.808 to -0.734 | <0.001 |
|  |  | RMSE | -0.051 days | -0.053 to -0.048 | <0.001 |
| Age <= 30 | ML(SG) vs. NR | Exact day | +0.292 pp | +0.068 to +0.507 | 0.009 |
|  |  | Within ±1 day | +0.508 pp | +0.250 to +0.763 | <0.001 |
|  |  | Within ±7 days | +2.171 pp | +1.947 to +2.362 | <0.001 |
|  |  | MSE | -1.568 days^2^ | -1.709 to -1.420 | <0.001 |
|  |  | RMSE | -0.102 days | -0.112 to -0.093 | <0.001 |
| Age <= 30 | ML(SG) vs. US | Exact day | -0.384 pp | -0.619 to -0.156 | <0.001 |
|  |  | Within ±1 day | -1.138 pp | -1.508 to -0.764 | <0.001 |
|  |  | Within ±7 days | -2.544 pp | -2.938 to -2.150 | <0.001 |
|  |  | MSE | +2.293 days^2^ | -1.080 to +4.154 | 0.095 |
|  |  | RMSE | +0.152 days | -0.071 to +0.277 | 0.095 |
| Age > 30 | ML vs. NR | Exact day | +0.312 pp | +0.103 to +0.537 | 0.008 |
|  |  | Within ±1 day | +0.621 pp | +0.385 to +0.863 | <0.001 |
|  |  | Within ±7 days | +1.761 pp | +1.567 to +1.956 | <0.001 |
|  |  | MSE | -1.733 days^2^ | -1.850 to -1.606 | <0.001 |
|  |  | RMSE | -0.114 days | -0.122 to -0.106 | <0.001 |
| Age > 30 | ML vs. US | Exact day | -0.111 pp | -0.362 to +0.158 | 0.420 |
|  |  | Within ±1 day | -0.285 pp | -0.736 to +0.128 | 0.163 |
|  |  | Within ±7 days | -0.721 pp | -1.160 to -0.291 | <0.001 |
|  |  | MSE | +2.311 days^2^ | +1.780 to +2.827 | <0.001 |
|  |  | RMSE | +0.155 days | +0.120 to +0.190 | <0.001 |
| Age > 30 | ML vs. ML(SG) | Exact day | +0.002 pp | -0.148 to +0.173 | 0.993 |
|  |  | Within ±1 day | +0.206 pp | +0.043 to +0.362 | 0.013 |
|  |  | Within ±7 days | +0.694 pp | +0.569 to +0.824 | <0.001 |
|  |  | MSE | -1.053 days^2^ | -1.117 to -0.988 | <0.001 |
|  |  | RMSE | -0.070 days | -0.074 to -0.065 | <0.001 |
| Age > 30 | ML(SG) vs. NR | Exact day | +0.310 pp | +0.090 to +0.529 | 0.006 |
|  |  | Within ±1 day | +0.415 pp | +0.182 to +0.634 | 0.002 |
|  |  | Within ±7 days | +1.066 pp | +0.884 to +1.246 | <0.001 |
|  |  | MSE | -0.680 days^2^ | -0.785 to -0.573 | <0.001 |
|  |  | RMSE | -0.045 days | -0.052 to -0.038 | <0.001 |
| Age > 30 | ML(SG) vs. US | Exact day | -0.113 pp | -0.366 to +0.143 | 0.414 |
|  |  | Within ±1 day | -0.492 pp | -0.927 to -0.086 | 0.024 |
|  |  | Within ±7 days | -1.415 pp | -1.853 to -0.987 | <0.001 |
|  |  | MSE | +3.364 days^2^ | +2.826 to +3.886 | <0.001 |
|  |  | RMSE | +0.225 days | +0.189 to +0.259 | <0.001 |
| BMI <= 18.5 | ML vs. NR | Exact day | -0.302 pp | -1.146 to +0.483 | 0.451 |
|  |  | Within ±1 day | +0.483 pp | -0.302 to +1.268 | 0.251 |
|  |  | Within ±7 days | +0.905 pp | +0.271 to +1.599 | 0.012 |
|  |  | MSE | -1.633 days^2^ | -2.043 to -1.229 | <0.001 |
|  |  | RMSE | -0.107 days | -0.133 to -0.080 | <0.001 |
| BMI <= 18.5 | ML vs. US | Exact day | +0.452 pp | -0.574 to +1.569 | 0.419 |
|  |  | Within ±1 day | -0.422 pp | -1.962 to +1.328 | 0.647 |
|  |  | Within ±7 days | -1.659 pp | -3.439 to +0.211 | 0.075 |
|  |  | MSE | +0.759 days^2^ | -1.551 to +3.105 | 0.544 |
|  |  | RMSE | +0.050 days | -0.102 to +0.203 | 0.546 |
| BMI <= 18.5 | ML vs. ML(SG) | Exact day | +0.271 pp | -0.332 to +0.845 | 0.381 |
|  |  | Within ±1 day | +0.181 pp | -0.483 to +0.875 | 0.612 |
|  |  | Within ±7 days | +1.207 pp | +0.664 to +1.780 | <0.001 |
|  |  | MSE | -1.501 days^2^ | -1.737 to -1.279 | <0.001 |
|  |  | RMSE | -0.098 days | -0.113 to -0.084 | <0.001 |
| BMI <= 18.5 | ML(SG) vs. NR | Exact day | -0.573 pp | -1.327 to +0.182 | 0.138 |
|  |  | Within ±1 day | +0.302 pp | -0.603 to +1.176 | 0.508 |
|  |  | Within ±7 days | -0.302 pp | -0.995 to +0.483 | 0.408 |
|  |  | MSE | -0.132 days^2^ | -0.580 to +0.302 | 0.563 |
|  |  | RMSE | -0.009 days | -0.038 to +0.020 | 0.564 |
| BMI <= 18.5 | ML(SG) vs. US | Exact day | +0.181 pp | -0.784 to +1.327 | 0.750 |
|  |  | Within ±1 day | -0.603 pp | -2.172 to +1.148 | 0.502 |
|  |  | Within ±7 days | -2.866 pp | -4.677 to -0.965 | 0.004 |
|  |  | MSE | +2.260 days^2^ | -0.064 to +4.594 | 0.060 |
|  |  | RMSE | +0.148 days | -0.004 to +0.303 | 0.058 |
| 18.5 < BMI <= 24.5 | ML vs. NR | Exact day | +0.321 pp | +0.105 to +0.521 | 0.002 |
|  |  | Within ±1 day | +0.482 pp | +0.279 to +0.685 | <0.001 |
|  |  | Within ±7 days | +1.721 pp | +1.552 to +1.894 | <0.001 |
|  |  | MSE | -1.519 days^2^ | -1.621 to -1.417 | <0.001 |
|  |  | RMSE | -0.100 days | -0.107 to -0.093 | <0.001 |
| 18.5 < BMI <= 24.5 | ML vs. US | Exact day | -0.305 pp | -0.533 to -0.095 | 0.011 |
|  |  | Within ±1 day | -0.687 pp | -1.032 to -0.344 | 0.002 |
|  |  | Within ±7 days | -1.706 pp | -2.048 to -1.339 | <0.001 |
|  |  | MSE | +3.402 days^2^ | +2.942 to +3.829 | <0.001 |
|  |  | RMSE | +0.229 days | +0.199 to +0.258 | <0.001 |
| 18.5 < BMI <= 24.5 | ML vs. ML(SG) | Exact day | +0.044 pp | -0.052 to +0.138 | 0.385 |
|  |  | Within ±1 day | +0.044 pp | -0.058 to +0.141 | 0.357 |
|  |  | Within ±7 days | +0.036 pp | -0.042 to +0.116 | 0.365 |
|  |  | MSE | -0.006 days^2^ | -0.034 to +0.023 | 0.633 |
|  |  | RMSE | +0.000 days | -0.002 to +0.001 | 0.633 |
| 18.5 < BMI <= 24.5 | ML(SG) vs. NR | Exact day | +0.277 pp | +0.060 to +0.468 | 0.009 |
|  |  | Within ±1 day | +0.438 pp | +0.234 to +0.628 | <0.001 |
|  |  | Within ±7 days | +1.685 pp | +1.514 to +1.853 | <0.001 |
|  |  | MSE | -1.513 days^2^ | -1.609 to -1.407 | <0.001 |
|  |  | RMSE | -0.100 days | -0.106 to -0.093 | <0.001 |
| 18.5 < BMI <= 24.5 | ML(SG) vs. US | Exact day | -0.349 pp | -0.574 to -0.136 | 0.003 |
|  |  | Within ±1 day | -0.731 pp | -1.071 to -0.386 | 0.002 |
|  |  | Within ±7 days | -1.743 pp | -2.068 to -1.385 | <0.001 |
|  |  | MSE | +3.408 days^2^ | +2.948 to +3.828 | <0.001 |
|  |  | RMSE | +0.230 days | +0.199 to +0.258 | <0.001 |
| 24.5 < BMI <= 30.0 | ML vs. NR | Exact day | +0.508 pp | +0.191 to +0.821 | 0.004 |
|  |  | Within ±1 day | +0.838 pp | +0.458 to +1.198 | <0.001 |
|  |  | Within ±7 days | +2.751 pp | +2.441 to +3.091 | <0.001 |
|  |  | MSE | -2.549 days^2^ | -2.781 to -2.337 | <0.001 |
|  |  | RMSE | -0.167 days | -0.182 to -0.153 | <0.001 |
| 24.5 < BMI <= 30.0 | ML vs. US | Exact day | -0.056 pp | -0.419 to +0.267 | 0.736 |
|  |  | Within ±1 day | -0.640 pp | -1.181 to -0.125 | 0.020 |
|  |  | Within ±7 days | -1.659 pp | -2.200 to -1.115 | <0.001 |
|  |  | MSE | +3.009 days^2^ | +2.286 to +3.685 | <0.001 |
|  |  | RMSE | +0.202 days | +0.154 to +0.247 | <0.001 |
| 24.5 < BMI <= 30.0 | ML vs. ML(SG) | Exact day | +0.099 pp | -0.073 to +0.280 | 0.277 |
|  |  | Within ±1 day | +0.063 pp | -0.109 to +0.238 | 0.503 |
|  |  | Within ±7 days | +0.393 pp | +0.241 to +0.541 | <0.001 |
|  |  | MSE | -0.914 days^2^ | -0.966 to -0.858 | <0.001 |
|  |  | RMSE | -0.060 days | -0.064 to -0.057 | <0.001 |
| 24.5 < BMI <= 30.0 | ML(SG) vs. NR | Exact day | +0.409 pp | +0.079 to +0.703 | 0.013 |
|  |  | Within ±1 day | +0.775 pp | +0.383 to +1.154 | <0.001 |
|  |  | Within ±7 days | +2.358 pp | +2.052 to +2.702 | <0.001 |
|  |  | MSE | -1.635 days^2^ | -1.868 to -1.416 | <0.001 |
|  |  | RMSE | -0.107 days | -0.122 to -0.092 | <0.001 |
| 24.5 < BMI <= 30.0 | ML(SG) vs. US | Exact day | -0.155 pp | -0.505 to +0.175 | 0.366 |
|  |  | Within ±1 day | -0.703 pp | -1.280 to -0.188 | 0.017 |
|  |  | Within ±7 days | -2.052 pp | -2.590 to -1.491 | <0.001 |
|  |  | MSE | +3.922 days^2^ | +3.207 to +4.623 | <0.001 |
|  |  | RMSE | +0.262 days | +0.214 to +0.309 | <0.001 |
| BMI > 30.0 | ML vs. NR | Exact day | +0.464 pp | -0.039 to +0.969 | 0.074 |
|  |  | Within ±1 day | +1.086 pp | +0.503 to +1.716 | 0.003 |
|  |  | Within ±7 days | +4.669 pp | +4.149 to +5.275 | <0.001 |
|  |  | MSE | -3.994 days^2^ | -4.421 to -3.582 | <0.001 |
|  |  | RMSE | -0.256 days | -0.284 to -0.229 | <0.001 |
| BMI > 30.0 | ML vs. US | Exact day | +0.016 pp | -0.520 to +0.559 | 0.960 |
|  |  | Within ±1 day | -0.417 pp | -1.244 to +0.323 | 0.301 |
|  |  | Within ±7 days | -0.354 pp | -1.260 to +0.551 | 0.467 |
|  |  | MSE | +1.463 days^2^ | +0.234 to +2.641 | 0.020 |
|  |  | RMSE | +0.096 days | +0.015 to +0.172 | 0.020 |
| BMI > 30.0 | ML vs. ML(SG) | Exact day | +0.220 pp | -0.055 to +0.496 | 0.124 |
|  |  | Within ±1 day | -0.079 pp | -0.370 to +0.205 | 0.598 |
|  |  | Within ±7 days | +0.779 pp | +0.559 to +1.016 | <0.001 |
|  |  | MSE | -1.471 days^2^ | -1.573 to -1.359 | <0.001 |
|  |  | RMSE | -0.095 days | -0.101 to -0.088 | <0.001 |
| BMI > 30.0 | ML(SG) vs. NR | Exact day | +0.244 pp | -0.244 to +0.717 | 0.322 |
|  |  | Within ±1 day | +1.165 pp | +0.582 to +1.826 | 0.002 |
|  |  | Within ±7 days | +3.889 pp | +3.330 to +4.480 | <0.001 |
|  |  | MSE | -2.523 days^2^ | -2.949 to -2.083 | <0.001 |
|  |  | RMSE | -0.161 days | -0.189 to -0.132 | <0.001 |
| BMI > 30.0 | ML(SG) vs. US | Exact day | -0.205 pp | -0.709 to +0.307 | 0.420 |
|  |  | Within ±1 day | -0.339 pp | -1.157 to +0.410 | 0.414 |
|  |  | Within ±7 days | -1.134 pp | -2.055 to -0.228 | 0.015 |
|  |  | MSE | +2.934 days^2^ | +1.705 to +4.122 | <0.001 |
|  |  | RMSE | +0.191 days | +0.111 to +0.268 | <0.001 |
| Nicotine Yes | ML vs. NR | Exact day | +0.202 pp | -0.202 to +0.617 | 0.312 |
|  |  | Within ±1 day | +0.844 pp | +0.385 to +1.250 | <0.001 |
|  |  | Within ±7 days | +2.528 pp | +2.168 to +2.884 | <0.001 |
|  |  | MSE | -2.587 days^2^ | -2.825 to -2.321 | <0.001 |
|  |  | RMSE | -0.167 days | -0.183 to -0.150 | <0.001 |
| Nicotine Yes | ML vs. US | Exact day | -0.346 pp | -0.746 to +0.074 | 0.108 |
|  |  | Within ±1 day | -0.676 pp | -1.323 to -0.074 | 0.034 |
|  |  | Within ±7 days | -1.689 pp | -2.434 to -0.938 | <0.001 |
|  |  | MSE | +2.155 days^2^ | +1.231 to +3.162 | <0.001 |
|  |  | RMSE | +0.142 days | +0.081 to +0.208 | <0.001 |
| Nicotine Yes | ML vs. ML(SG) | Exact day | -0.010 pp | -0.212 to +0.188 | 0.955 |
|  |  | Within ±1 day | -0.059 pp | -0.272 to +0.134 | 0.597 |
|  |  | Within ±7 days | +0.602 pp | +0.430 to +0.790 | <0.001 |
|  |  | MSE | -1.146 days^2^ | -1.218 to -1.075 | <0.001 |
|  |  | RMSE | -0.075 days | -0.079 to -0.070 | <0.001 |
| Nicotine Yes | ML(SG) vs. NR | Exact day | +0.212 pp | -0.217 to +0.598 | 0.293 |
|  |  | Within ±1 day | +0.904 pp | +0.459 to +1.323 | <0.001 |
|  |  | Within ±7 days | +1.926 pp | +1.560 to +2.286 | <0.001 |
|  |  | MSE | -1.442 days^2^ | -1.679 to -1.164 | <0.001 |
|  |  | RMSE | -0.093 days | -0.109 to -0.075 | <0.001 |
| Nicotine Yes | ML(SG) vs. US | Exact day | -0.336 pp | -0.741 to +0.074 | 0.120 |
|  |  | Within ±1 day | -0.617 pp | -1.279 to +0.015 | 0.060 |
|  |  | Within ±7 days | -2.291 pp | -3.027 to -1.550 | <0.001 |
|  |  | MSE | +3.301 days^2^ | +2.354 to +4.329 | <0.001 |
|  |  | RMSE | +0.217 days | +0.156 to +0.284 | <0.001 |
| Nicotine No | ML vs. NR | Exact day | +0.275 pp | +0.100 to +0.458 | 0.004 |
|  |  | Within ±1 day | +0.613 pp | +0.429 to +0.811 | <0.001 |
|  |  | Within ±7 days | +2.062 pp | +1.899 to +2.214 | <0.001 |
|  |  | MSE | -1.991 days^2^ | -2.088 to -1.888 | <0.001 |
|  |  | RMSE | -0.131 days | -0.138 to -0.124 | <0.001 |
| Nicotine No | ML vs. US | Exact day | -0.178 pp | -0.361 to +0.011 | 0.066 |
|  |  | Within ±1 day | -0.578 pp | -0.852 to -0.300 | <0.001 |
|  |  | Within ±7 days | -1.585 pp | -1.902 to -1.269 | <0.001 |
|  |  | MSE | +2.765 days^2^ | +2.400 to +3.185 | <0.001 |
|  |  | RMSE | +0.186 days | +0.162 to +0.214 | <0.001 |
| Nicotine No | ML vs. ML(SG) | Exact day | +0.019 pp | -0.071 to +0.110 | 0.695 |
|  |  | Within ±1 day | +0.110 pp | +0.030 to +0.198 | 0.017 |
|  |  | Within ±7 days | +0.377 pp | +0.301 to +0.447 | <0.001 |
|  |  | MSE | -0.835 days^2^ | -0.861 to -0.808 | <0.001 |
|  |  | RMSE | -0.055 days | -0.057 to -0.054 | <0.001 |
| Nicotine No | ML(SG) vs. NR | Exact day | +0.256 pp | +0.083 to +0.435 | 0.007 |
|  |  | Within ±1 day | +0.502 pp | +0.305 to +0.702 | <0.001 |
|  |  | Within ±7 days | +1.685 pp | +1.525 to +1.837 | <0.001 |
|  |  | MSE | -1.156 days^2^ | -1.257 to -1.046 | <0.001 |
|  |  | RMSE | -0.076 days | -0.083 to -0.069 | <0.001 |
| Nicotine No | ML(SG) vs. US | Exact day | -0.197 pp | -0.381 to +0.000 | 0.041 |
|  |  | Within ±1 day | -0.688 pp | -0.977 to -0.421 | <0.001 |
|  |  | Within ±7 days | -1.962 pp | -2.284 to -1.646 | <0.001 |
|  |  | MSE | +3.599 days^2^ | +3.229 to +4.022 | <0.001 |
|  |  | RMSE | +0.242 days | +0.217 to +0.270 | <0.001 |

Note: Differences were calculated within the same subgroup-specific independent test set and estimated using paired non-parametric bootstrap resampling with 1,000 replications. For exact-day, ±1-day, and ±7-day accuracy, positive differences indicate higher accuracy for the first method; for MSE and RMSE, negative differences indicate lower prediction error for the first method. Sample sizes are: Parity = 0 (N = 51,579); Parity ≥ 1 (N = 47,607); Age ≤ 30 (N = 67,493); Age > 30 (N = 53,278); BMI ≤ 18.5 (N = 3,315); 18.5 < BMI ≤ 24.5 (N = 74,431); 24.5 < BMI ≤ 30.0 (N = 30,317); BMI > 30.0 (N = 12,702); Nicotine Yes (N = 20,252); Nicotine No (N = 100,517). CI, confidence interval; pp, percentage points; ML, primary machine-learning model; ML(SG), subgroup-specific machine-learning model; NR, Naegele’s rule; US, ultrasound-based dating; MSE, mean squared error; RMSE, root mean squared error. Data source: Swedish Medical Birth Register (*Medicinska födelseregistret*), 2008–2017.
